# A Co-expression Analysis of the Placental Transcriptome in Association With Maternal Pre-pregnancy BMI and Newborn Birth Weight

**DOI:** 10.3389/fgene.2019.00354

**Published:** 2019-04-29

**Authors:** Bianca Cox, Maria Tsamou, Karen Vrijens, Kristof Y. Neven, Ellen Winckelmans, Theo M. de Kok, Michelle Plusquin, Tim S. Nawrot

**Affiliations:** ^1^Center for Environmental Sciences, Hasselt University, Hasselt, Belgium; ^2^Department of Toxicogenomics, Maastricht University, Maastricht, Netherlands; ^3^Department of Public Health, Environment and Health Unit, Leuven University (KU Leuven), Leuven, Belgium

**Keywords:** maternal pre-pregnancy BMI, birth weight, placenta, transcriptome, microarray, WGCNA

## Abstract

Maternal body mass index (BMI) before pregnancy is known to affect both fetal growth and later-life health of the newborn, yet the implicated molecular mechanisms remain largely unknown. As the master regulator of the fetal environment, the placenta is a valuable resource for the investigation of processes involved in the developmental programming of metabolic health. We conducted a genome-wide placental transcriptome study aiming at the identification of functional pathways representing the molecular link between maternal BMI and fetal growth. We used RNA microarray (Agilent 8 × 60 K), medical records, and questionnaire data from 183 mother-newborn pairs from the ENVIR*ON*AGE birth cohort study (Flanders, Belgium). Using a weighted gene co-expression network analysis, we identified 17 correlated gene modules. Three of these modules were associated with both maternal pre-pregnancy BMI and newborn birth weight. A gene cluster enriched for genes involved in immune response and myeloid cell differentiation was positively associated with maternal BMI and negatively with low birth weight. Two other gene modules, upregulated in association with maternal BMI as well as birth weight, were involved in processes related to organ and tissue development, with blood vessel morphogenesis and extracellular matrix structure as top Gene Ontology terms. In line with this, erythrocyte-, angiogenesis-, and extracellular matrix-related genes were among the identified hub genes. The association between maternal BMI and newborn weight was significantly mediated by gene expression for 5 of the hub genes (*FZD4*, *COL15A1*, *GPR124*, *COL6A1*, and *COL1A1*). As some of the identified hub genes have been linked to obesity in adults, our observation in placental tissue suggests that biological processes may be affected from prenatal life onwards, thereby identifying new molecular processes linking maternal BMI and fetal metabolic programming.

## Introduction

In 2016, 39% of the world’s adult population was overweight, and 13% was obese (Abarca-Gómez et al., 2017). In the context of fetal programming, it is well-accepted that an obesogenic intrauterine environment has long-lasting effects on the fetus. A high maternal body mass index (BMI) is a risk factor for adverse birth outcomes and infant death (Aune et al., 2014; Marchi et al., 2015), and offspring of obese mothers have a higher risk of developing obesity, diabetes, and cardiovascular diseases in later life (Gaillard, 2015).

Recent advances in molecular technologies suggest that the placenta is the master regulator of the fetal environment, representing a higher level of control of fetal programming compared to other tissues (Konkel, 2016). The placenta plays a critical role in nutrient and waste transfer, endocrine secretion, immunological protection, and xenobiotic detoxification (Burton et al., 2016). In addition, the placenta can undergo major structural and functional adaptations in order to protect the fetus from environmental stressors. However, if the organ’s capacity for adaptation is exceeded or if placental function is impaired, the intrauterine environment might be perturbed and fetal development could be affected, with potential adverse consequences for later-life health.

Despite the central role of the placenta in fetal programming, only a limited number of studies have assessed the genome-wide effect of maternal weight on the human placental transcriptome (Saben et al., 2014; Altmäe et al., 2017). Studies assessing placental transcriptome profiles related to fetal growth mostly focused on intrauterine growth restriction (Nishizawa et al., 2011; Madeleneau et al., 2015), often in the context of pre-eclampsia (Kleinrouweler et al., 2013), whereas genome-scale studies linking placental gene expression with excessive fetal growth are scarce (Sabri et al., 2014; Ahlsson et al., 2015; Sober et al., 2015; Song et al., 2018). Most of the previous studies used a univariate differential expression analysis approach, typically comparing a limited number of adverse with normal phenotypes. Methods taking into account the correlations between genes, such as weighted gene co-expression network analysis (WGCNA) (Zhao et al., 2010), have been proposed to facilitate the identification of genes with similar functions, thereby providing a systematic understanding of molecular mechanisms underlying biological processes.

The identification of a placental gene expression signature related to maternal BMI as well as fetal growth may provide new insights into the molecular mechanisms underlying the intrauterine programming of metabolic health. To the best of our knowledge, no study so far has looked at the overlap between placental transcriptome profiles related to maternal pre-pregnancy BMI and fetal growth. Using WGCNA, we identified placental co-expressed gene modules and hub genes associated with both maternal pre-pregnancy BMI and weight of the newborn and we investigated whether these modules and hub genes mediated the association between pre-pregnancy BMI and newborn weight.

## Materials and Methods

### Study Population

Within the framework of the ongoing Belgian birth cohort ENVIR*ON*AGE (ENVIRonmental influence *ON* early AGEing), mother-newborn pairs were recruited upon arrival for delivery at the East-Limburg Hospital in Genk, Flanders (Janssen et al., 2017). Procedures were approved by the Ethical Committee of Hasselt University and the East-Limburg Hospital and recruitment was carried out according to the Helsinki declaration. Mothers without a planned cesarean section and who were able to fill out a questionnaire in Dutch, were eligible for participation. For this study, we used a subsample of 195 mother-newborn pairs recruited between January 2014 and April 2017. Samples with missing covariate information (*n* = 5), a birth weight below 1000 g (*n* = 1), or low quality of extracted RNA (RNA integrity number below 6) (*n* = 6), were excluded from the analysis, resulting in a final sample size of 183.

Information on variables such as gestational age, birth weight, maternal pre-pregnancy weight, height, and weight before delivery were retrieved from the medical records of the hospital. Gestational age was estimated based on the mother’s last menstrual period in combination with ultrasound data. Maternal height and weight were measured at the first antenatal visit (weeks 7–9 of gestation) wearing no shoes and light clothes. Pre-pregnancy BMI was calculated as weight in kilograms divided by the square of height in meters and categorized into four groups: underweight (below 18.5 kg/m^2^), normal weight (18.5–24.9 kg/m^2^), overweight (25.0–29.9 kg/m^2^), and obese (30.0 kg/m^2^ or above). Maternal pregnancy weight gain was calculated from the pre-pregnancy weight and the weight measured on admission to the delivery ward. Low birth weight was defined as a birth weight below the 10^th^ percentile of the sample (2643 g), and high birth weight as a birth weight above the 90^th^ percentile (3963 g).

Detailed information about socio-demographic and lifestyle factors such as maternal age, smoking status during pregnancy, parity, and newborn’s ethnicity were obtained from questionnaires. Maternal smoking status was defined as never smokers, past smokers (quit smoking cigarettes before pregnancy), and current smokers (smoked cigarettes during pregnancy). Parity was categorized into 1, 2, and ≥3 children. Classification of ethnicity is based on the native country of the neonates’ grandparents as either European (at least two grandparents were European) or non-European (at least three grandparents were of non-European origin).

### Sample Collection and RNA Isolation

Placental tissue was collected within 1 h after delivery. Four standardized biopsies were taken from the fetal side, at fixed locations across the middle point of the placenta around 4 cm distance from the umbilical cord (Janssen et al., 2017). The collected biopsies were stored in RNA *later* (Thermo Fisher Scientific, Waltham, MA, United States) at 4°C for at least 12 h and maximally 24 h, followed by storage at -20°C. Total RNA was extracted from one placental tissue biopsy using the miRNeasy mini kit (Qiagen, Venlo, Netherlands) according to the manufacturer’s protocol. RNase-Free DNase treatment was performed on RNA samples according to the manufacturer’s instructions (Qiagen, Venlo, Netherlands). RNA quantity and purity was assessed by spectrophotometry (Nanodrop 1000, Isogen Life Science, De Meern, Netherlands) and RNA integrity by Agilent 2100 Bioanalyzer (Agilent Technologies, Amstelveen, Netherlands).

### Microarray Preparation, Hybridization, and Preprocessing

0.2 μg total RNA was used to synthesize fluorescent cyanine-3-labeled cRNA following the Agilent one-color Quick-Amp labeling protocol (Agilent Technologies) and hybridized onto Agilent Whole Human Genome 8 × 60 K microarrays. Microarray signals were detected using the Agilent DNA G2505C Microarray Scanner (Agilent Technologies). Scan images were converted into TXT files using the Agilent Feature Extraction Software (Version 10.7.3.1, Agilent Technologies, Amstelveen, Netherlands). An in-house developed quality control pipeline in R software was used to preprocess raw data as follows: local background correction, omission of controls, flagging of bad spots and spots with too low intensity, log2 transformation and quantile normalization using arrayQC. More information about the flagging and the R-scripts of the pipeline are available at https://github.com/BiGCAT-UM/arrayQC_Module. Further preprocessing included removal of probes showing > 30% flagged data, merging of replicate probes based on the median, and imputation of missing values by means of K-nearest neighbor imputation (*K* = 15). Batch effects were corrected for by using an empirical Bayes method (ComBat) (Johnson et al., 2007). For genes with multiple probes, the probe with the largest interquartile range was selected. From the resulting 18,847 probes, only those with expression levels above 6 (in the log2 scale) in a minimum of 30 samples were kept, leaving 14,040 genes for further analysis. Data are available via NCBI Gene Expression Omnibus (GEO) with the Accession No. GSE128381.

### Statistical Analysis

The association between maternal pre-pregnancy BMI (as a continuous and as categorical variable) and birth weight was assessed through a linear regression model adjusting for date of delivery, newborn sex, gestational age, ethnicity, parity, maternal age, maternal smoking, and weight gain during pregnancy.

First, we assessed the associations of transcripts levels (of 14,040 genes) with maternal pre-pregnancy BMI and with birth weight using univariate models, correcting for the same variables as above. We adjusted for multiple testing by controlling the Benjamini–Hochberg false discovery rate (FDR) at 5%.

Then we constructed a gene co-expression network by using the WGCNA package (Langfelder and Horvath, 2008) in R, following the general WGCNA guidelines (Zhang and Horvath, 2005). Briefly, pairwise Pearson correlation coefficients between all (*n* = 14,040) genes were calculated to generate a signed similarity. A weighted adjacency matrix was obtained by raising the signed similarity matrix to a power β, which was set to 14 after a sensitivity analysis of scale-free topology (*R*^2^ > 0.9). The adjacency matrix was then converted to a topological overlapping matrix (TOM) network, which was used as input for a hierarchical clustering analysis. Finally, modules were identified by implementing a dynamic tree cutting algorithm on the TOM-based dendrogram, using the parameters deepSplit = 2 and minClusterSize = 30. The resulting gene clusters (modules) get a color name as identifier, with *gray* denoting background genes outside of modules. The module eigengene (ME), calculated as the eigenvector associated with the first principal component of the expression matrix, serves as a summary measure for the module. A cut height of 0.35 was used to merge modules with a correlation between MEs of 0.65 or greater.

To identify gene clusters associated with traits such as maternal pre-pregnancy BMI, we used linear regression models treating the MEs as dependent variables and the traits as independent variables, correcting for the same variables as above. Maternal BMI and newborn birth weight were modeled as continuous and as categorical variables in order to capture potential non-linear associations. Normal pre-pregnancy BMI and normal birth weight were used as reference categories. Regression coefficients were expressed as partial Pearson correlations (with *P*-values), using the ppcor package in R (Kim, 2015). Modules of interest were those that were significantly correlated with one of the maternal BMI variables (continuous variable or one of the BMI categories) as well as with one of the birth weight variables (continuous variable or low birth weight or high birth weight). The *P*-value cut-off for selecting modules of interest was set at 0.1.

Modules of interest were further characterized by gene ontology (GO) and pathway enrichment analyses. Overrepresented GO biological processes and Kyoto Encyclopedia of Genes and Genomes (KEGG) pathways inside each module were identified by using WebGestalt (Zhang et al., 2005; Wang et al., 2013), considering a FDR < 0.05 as the criterion for statistical significance after Benjamini–Hochberg correction for multiple testing. The REVIGO tool (Supek et al., 2011) was used to filter out redundant GO terms and to visualize enrichment analysis results.

For the modules of interest, highly connected intramodular genes (hub genes) related to maternal pre-pregnancy BMI and birth weight were selected based on two criteria: (1) the module membership (MM), calculated as the Pearson correlation between the expression of a gene and the ME, and (2) the significance of the partial Pearson correlations between expression levels of individual genes and the traits of interest [BMI, BMI categories (underweight, overweight, obese), birth weight, birth weight categories (low and high birth weight)]. Partial Pearson correlations (with *P*-values) were obtained as described for the MEs and were corrected for the same set of variables. Hub genes were defined as genes with |MM| ≥ 0.8 and *P* < 0.05 for at least one maternal BMI variable and at least one birth weight variable. In separate sensitivity analyses, associations of traits of interest with MEs and hub genes were tested after excluding non-European newborns (*n* = 26), mothers with gestational diabetes (*n* = 6), and mothers with gestational hypertension (*n* = 8) from the study population.

To investigate expression of identified modules and genes as a potential molecular link between maternal pre-pregnancy BMI and newborn birth weight, we performed mediation analyses with pre-pregnancy BMI as independent causal variable and birth weight as outcome. The mediating effect of modules and hub genes was tested using the default quasi-Bayesian Monte Carlo method and bootstrap simulation (10000 simulations) from the R mediation package (Tingley et al., 2014).

## Results

General characteristics of our study population (*n* = 183) are provided in Table 1. 4.4% of the mothers was underweight, 26.2% was overweight and 14.8% was obese before pregnancy. Our study population included 24 (13.1%) preterm births (gestational age below 37 weeks). Most of the low birth weight babies (16 out of 19) were preterm and all high birth weight babies had a gestational age above 38 weeks. Our sample was representative for all deliveries in Flanders (Cox et al., 2013) with respect to newborn birth weight [average (10^th^–90^th^ percentile) = 3328 (2643–3963) gram compared to 3360 (2740–3965) gram] and characteristics such as maternal age, parity, sex, and ethnicity (Supplementary Table S1).

**Table 1:** Characteristics of study population (*n* = 183).

Characteristics	Mean ± SD/frequency (%)
**Mother**	
Age, *years*	29.9 ± 4.4
Pre-pregnancy BMI, *kg/m^2^∗^^*	25.0 ± 5.2
Underweight	8 (4.4)
Normal weight	100 (54.6)
Overweight	48 (26.2)
Obese	27 (14.8)
Gestational weight gain, *kg*	13.5 ± 5.7
Smoking status	
Never-smoker	123 (67.2)
Past-smoker	46 (25.2)
Current smoker	14 (7.6)
Parity	
*1*	89 (48.6)
*2*	71 (38.8)
*≥3*	23 (12.6)
Gestational diabetes	6 (3.3)
Gestational hypertension	8 (4.4)
**Newborn**	
Boys	95 (51.9)
Gestational age, *weeks*	38.9 ± 2.1
Birth weight, *g*^†^	3,328 ± 532
Low	19 (10.0)
Normal	145 (80.0)
High	19 (10.0)
European ethnicity^#^	157 (85.8)

^∗^*Pre-pregnancy BMI was coded as underweight (below 18.5 kg/m^*2*^), normal weight (18.5–24.9 kg/m^*2*^), overweight (25.0–29.9 kg/m^*2*^), and obese (30.0 kg/m^*2*^ or above). ^†^Low birth weight was defined as a birth weight below the 10^*th*^ percentile of the sample (<2643 g), normal weight as a birth weight between the 10^*th*^ and 90^*th*^ percentile (2643–3963 g) and high birth weight as a birth weight above the 90^*th*^ percentile (>3963 g). ^#^Newborns were classified as European when at least two grandparents were European, and non-European when at least three grandparents were of non-European origin*.

A histogram of birth weight and a scatterplot of birth weight versus maternal pre-pregnancy BMI with unadjusted and adjusted regression lines are presented in Supplementary Figure S1. Birth weight was positively associated with maternal pre-pregnancy BMI and was estimated to be 13.3 g (95% confidence interval [CI]: 3.0, 23.6 g) higher for a 1 kg/m^2^ higher maternal BMI. Compared with newborns of normal weight mothers, birth weight of newborns from underweight and overweight mothers was not significantly different (-46.7 g [95% CI: -305.4, 212.0 g] and 54.6 g [95% CI: -68.4, 177.7 g], respectively), but birth weight of those from obese mothers was significantly higher (202.2 g, 95% CI: 45.4, 359.0 g). The estimated increase in birth weight for a 1 kg increase in maternal weight gain during pregnancy was 16.7 g (95% CI: 7.2, 26.2 g).

### Univariate Models

In models assessing the associations between transcripts levels and maternal pre-pregnancy BMI or birth weight, none of 14,040 genes survived Benjamini–Hochberg correction for multiple testing. Unadjusted *P*-values for maternal BMI and birth weight were < 0.05 for 832 and 745 genes respectively, with an overlap of 101 genes (Supplementary Table S2). For all of the overlapping genes, the direction of the association with maternal BMI and with birth weight was consistent, with 75 of them being upregulated in association with both variables and 26 being downregulated in association with both.

### Weighted Gene Co-expression Network Analysis

**Figure 1:**
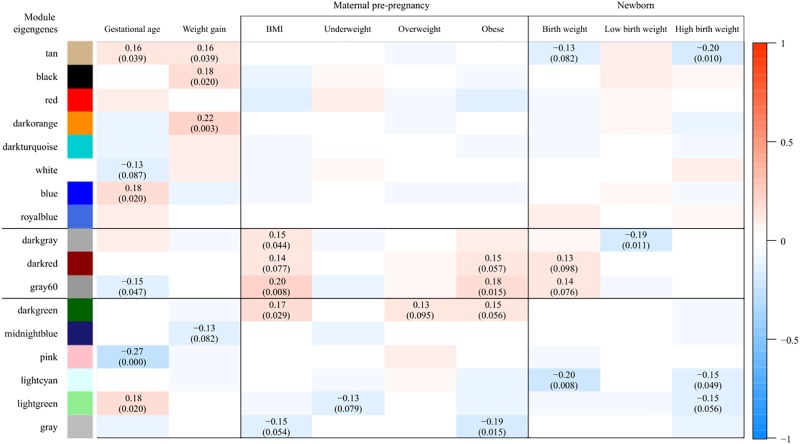
Associations between module eigengenes (ME, in rows) and traits (in columns). Colors indicate the strength and the direction of the correlation according to the color legend. The numbers represent the partial Pearson correlations with corresponding *P*-values in parenthesis (only those with *P* < 0.1 are shown). Partial correlations were obtained from models adjusted for date of delivery, newborn sex, gestational age, ethnicity, parity, maternal age, maternal smoking, and weight gain during pregnancy. Modules found to be associated (*P* < 0.1, corresponding to *r* ≥ 0.13) with at least one of the maternal BMI variables and at least one of the birth weight variables were *darkgray*, *darkred*, *gray60*, and *lightgreen*. As the correlations observed in the *lightgreen* module were driven by one specific observation, only the *darkgray*, *darkred*, and *gray60* modules were considered for further analyses.

Weighted gene co-expression network analysis identified 17 co-expressed gene modules (Supplementary Figure S2). The *gray* module, consisting of genes not attributed to any module, contained 9 genes. The size of other modules ranged from 48 to 5298 genes. A heatmap of the module-trait associations, presenting the partial Pearson correlations between MEs and traits, is shown in Figure 1. Modules found to be associated (*P* < 0.1, corresponding to *r* ≥ 0.13) with at least one of the maternal BMI variables and at least one of the birth weight variables were *darkgray* (*n* = 69 genes), *darkred* (*n* = 1091 genes), *gray60* (*n* = 204 genes), and *lightgreen* (*n* = 451 genes). The *darkgray* module showed a positive correlation with maternal pre-pregnancy BMI (*r* = 0.15, *P* = 0.044) and a negative correlation with low birth weight (*r* = -0.19, *P* = 0.011). The *darkred* module was positively associated with maternal BMI (*r* = 0.14, *P* = 0.077), with maternal obesity (*r* = 0.15, *P* = 0.06), and with birth weight (*r* = 0.13, *P* = 0.098). Also *gray60* was positively associated with maternal BMI (*r* = 0.20, *P* = 0.008), maternal obesity (*r* = 0.18, *P* = 0.015), and birth weight (*r* = 0.14, *P* = 0.076). The *lightgreen* module showed a negative correlation with maternal underweight (*r* = -0.13, *P* = 0.079), as well as with high birth weight (*r* = -0.15, *P* = 0.056).

We checked the robustness of the above associations by excluding underweight mothers with a high birth weight baby (*n* = 1) and overweight mothers with a low birth weight baby (*n* = 4) from the partial correlation analysis (our sample did not contain obese mothers with a low birth weight baby). We noticed that the correlations observed in the *lightgreen* module were driven by the one underweight mother with a high birth weight baby (both *P* > 0.1 after excluding this observation from the analysis). Exclusion of this particular observation did not alter the associations observed in the *darkgray*, *darkred*, and *gray60* modules, neither did the exclusion of the four overweight mothers having a newborn with low birth weight. Consequently, only the *darkgray*, *darkred*, and *gray60* modules were considered for further analyses.

### GO and Pathway Enrichment Analyses

**Figure 2:**
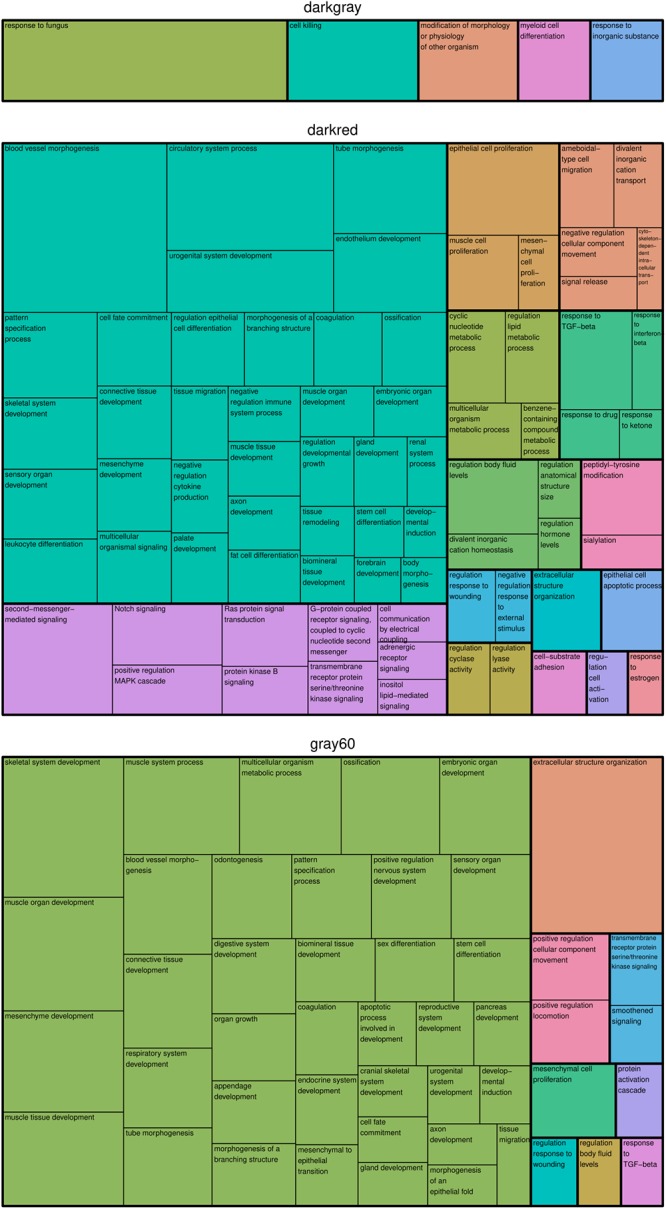
Treemap of GO biological processes enriched in the placental microarray modules of interest. REVIGO was used to remove redundant GO terms and to join the cluster representatives (the single rectangles) into superclusters (represented by different colors). The size of each rectangle reflects the FDR value of the GO term (larger for lower FDR).

**Figure 3:**
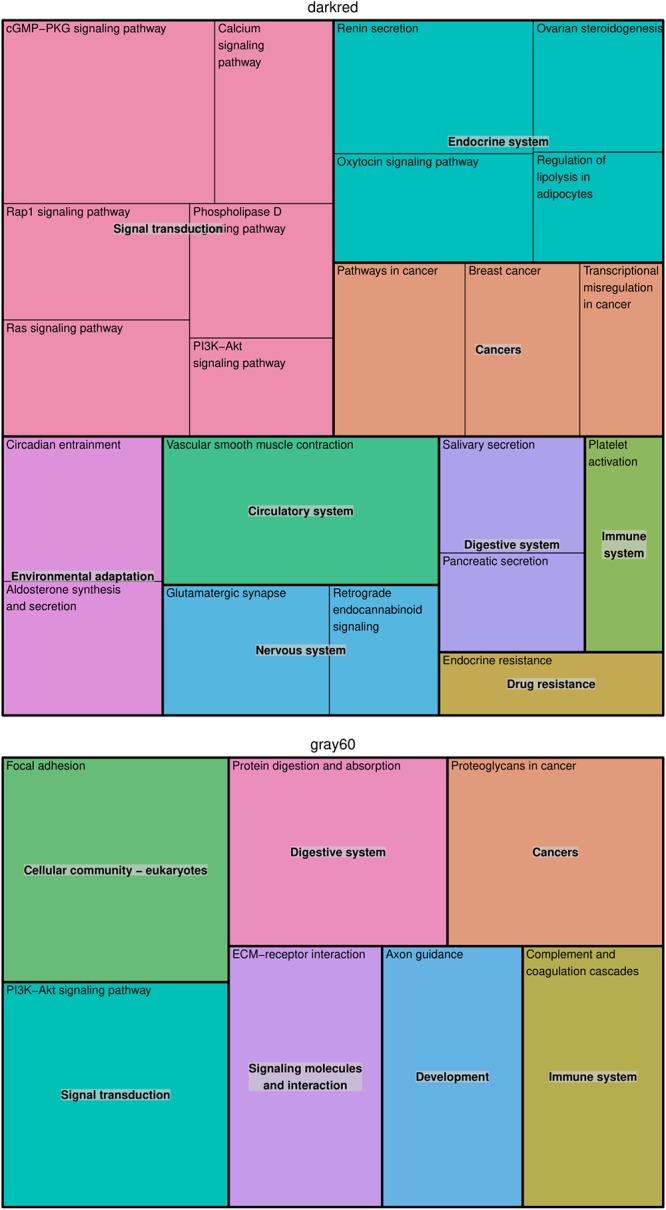
Treemap of KEGG pathways enriched in the placental microarray modules of interest. The KEGG hierarchy was used to join the pathways (the single rectangles) into superclusters (represented by different colors). The size of each rectangle reflects the FDR value of the pathway (larger for lower FDR).

In the modules *darkgray*, *darkred*, and *gray60*, we found 7, 110, and 56 enriched GO biological processes (Supplementary Table S3) and 0, 22, and 7 enriched KEGG pathways (Supplementary Table S4), respectively. Redundant GO terms were removed by REVIGO and results are summarized by means of a treemap (Figure 2). The five non-redundant GO biological processes in the *darkgray* module were response to fungus, cell killing, modification of morphology or physiology of other organism, myeloid cell differentiation, and response to inorganic substance, but no enriched KEGG pathways were found. The *darkred* and *gray60* modules were close to each other in the hierarchical clustering (Supplementary Figure S2), which is reflected in the overlap in enriched GO terms (28 common processes). Most of the biological processes in the *darkred* module were related to organ and tissue development (35 out of 76 enriched processes), with blood vessel morphogenesis as top GO term (lowest FDR). The most enriched KEGG pathway was vascular smooth muscle contraction, and other pathways involved signal transduction (six pathways), endocrine system (four pathways), cancer (three pathways), and environmental adaptation (two pathways) (Figure 3). Also in the *gray60* module, the majority of GO biological processes (37 out of 47) were related to organ and tissue development, with extracellular structure organization as the most enriched process. The *gray60* module contained seven enriched KEGG pathways, including PI3K-Akt signaling, focal adhesion, protein digestion and absorption, and extracellular matrix receptor interaction.

### Intramodular Hub Genes

Hub genes for the modules of interest were selected according to the criteria: |MM| ≥ 0.8 and significantly (*P* < 0.05) correlated with at least one maternal BMI variable and at least one birth weight variable (Table 2). Actual *P*-values of the partial correlations with BMI and birth weight variables are presented in Supplementary Table S5. In the *darkgray* module, we found five hub genes positively correlated with maternal BMI and negatively correlated with low birth weight: *XLOC_000346*, *AHSP*, *XLOC_013489*, *SLC4A1*, and *HBQ1*. The hub genes in the *darkred* module (*FZD4*, *CDC42EP2*, *COL15A1*, *TBXA2R*, *EHD2*, *GPR124*, *VIM*, *EFEMP2*, and *TBX2*) and in the *gray60* module (*COL8A2*, *MATN2*, *KANK2*, *COL6A1*, *TRPC1*, *COL16A1*, *COL3A1*, *RUNX1T1*, *AEBP1*, *COL1A1*, and *RGS11*) showed a positive correlation with maternal BMI and/or obesity, as well as with birth weight. *TRPC1* and *COL16A1* in the *gray60* module were also negatively correlated with maternal underweight. Fourteen of these genes (*FZD4*, *COL15A1*, *TBXA2R*, *EHD2*, *GPR124*, *COL8A2*, *MATN2*, *COL6A1*, *TRPC1*, *COL16A1*, *COL3A1*, *RUNX1T1*, *AEBP1*, and *COL1A1*) were also picked up in the univariate models (unadjusted *P*-values for maternal BMI and birth weight < 0.05), but FDR values were > 0.05.

**Table 2:** Hub genes for the modules of interest, defined as |MM| ≥ 0.8 and a significant partial correlation with at least one maternal BMI variable and at least one birth weight variable.

				Partial correlation		
			Maternal pre-pregnancy				Newborn		
Gene symbol	Gene name	MM	BMI	Under weight	Over weight	Obese	BW	Low BW	High BW
***Darkgray* module**								
*XLOC_000346*		0.85	0.17*	-0.02	0.02	0.16*	0.04	-0.16*	0.02
*AHSP*	Alpha hemoglobin stabilizing protein	0.88	0.17*	-0.05	0.00	0.12	0.04	-0.18*	-0.02
*XLOC_013489*		0.92	0.17*	-0.01	0.01	0.14	0.02	-0.18*	-0.05
*SLC4A1*	Solute carrier family 4 member 1 (Diego blood group)	0.91	0.15*	-0.04	-0.01	0.12	0.07	-0.19*	-0.04
*HBQ1*	Hemoglobin subunit theta 1	0.92	0.15*	-0.02	-0.04	0.14	0.02	-0.20**	-0.02
***Darkred* module**								
*FZD4*	Frizzled class receptor 4	0.83	0.24**	-0.03	0.10	0.25**	0.20**	-0.06	0.05
*CDC42EP2*	CDC42 effector protein 2	0.84	0.18*	-0.10	0.07	0.15	0.16*	-0.12	0.02
*COL15A1*	Collagen type XV alpha 1 chain	0.84	0.18*	-0.03	0.08	0.17*	0.20**	-0.08	0.00
*TBXA2R*	Thromboxane A2 receptor	0.81	0.18*	-0.05	0.12	0.13	0.16*	-0.02	0.04
*EHD2*	EH domain containing 2	0.89	0.16*	-0.04	0.04	0.18*	0.17*	-0.03	0.03
*GPR124*	G-protein coupled receptor 124	0.89	0.16*	0.00	0.05	0.18*	0.22**	-0.05	0.04
*VIM*	Vimentin	0.90	0.15*	-0.03	0.12	0.16*	0.17*	-0.05	0.04
*EFEMP2*	EGF containing fibulin extracellular matrix protein 2	0.88	0.15*	-0.03	0.11	0.13	0.16*	-0.06	0.04
*TBX2*	T-box 2	0.83	0.12	0.02	0.07	0.19*	0.18*	-0.07	0.03
***Gray60* module**								
*COL8A2*	Collagen type VIII alpha 2 chain	0.88	0.24**	-0.11	0.04	0.21**	0.16*	0.01	0.02
*MATN2*	Matrilin 2	0.86	0.24**	-0.09	0.10	0.19*	0.18*	-0.09	-0.01
*KANK2*	KN motif and ankyrin repeat domains 2	0.84	0.19*	-0.10	0.04	0.18*	0.17*	-0.05	0.01
*COL6A1*	Collagen type VI alpha 1 chain	0.83	0.18*	-0.07	0.06	0.18*	0.20**	-0.07	0.04
*TRPC1*	Transient receptor potential cation channel subfamily C member 1	0.81	0.18*	-0.19*	0.14	0.12	0.15*	-0.12	-0.01
*COL16A1*	Collagen type XVI alpha 1 chain	0.83	0.17*	-0.18*	-0.02	0.13	0.17*	-0.05	-0.04
*COL3A1*	Collagen type III alpha 1 chain	0.83	0.17*	-0.12	0.06	0.13	0.16*	-0.13	-0.05
*RUNX1T1*	RUNX1 translocation partner 1	0.86	0.16*	-0.02	0.07	0.20**	0.18*	-0.05	-0.03
*AEBP1*	AE binding protein 1	0.84	0.16*	-0.08	0.03	0.18*	0.16*	-0.08	-0.08
*COL1A1*	Collagen type I alpha 1 chain	0.82	0.16*	-0.09	0.08	0.12	0.22**	-0.07	0.07
*RGS11*	Regulator of G-protein signaling 11	0.81	0.13	-0.08	0.05	0.15*	0.24**	-0.08	0.00

^∗^*P-value of partial correlation < 0.05; ^∗∗^*P*-value of partial correlation < 0.01; MM, module membership; BW, birth weight*.

### Sensitivity Analyses

Associations between traits of interest and MEs or hub genes were assessed after excluding non-European newborns (Supplementary Table S6), mothers with gestational diabetes (Supplementary Table S7), and mothers with gestational hypertension (Supplementary Table S8) from the analysis. Although some *P*-values for correlations with MEs became larger than 0.1, correlation coefficients changed only slightly and associations with hub genes mostly remained significant (*P* < 0.05). The exclusion of mothers with hypertension, however, resulted in smaller and non-significant partial correlations with maternal BMI in the *darkgray* module, both for the ME as for the five hub genes.

### Mediation Analyses

Finally we tested whether the identified modules and genes mediated the association between maternal pre-pregnancy BMI and newborn birth weight (Supplementary Table S9). Although the indirect (mediating) effects of MEs were not significant, many of the hub genes from the *darkred* and *gray60* modules showed some evidence for mediation (*P*-value of the indirect effect < 0.1). The proportion of mediation (indirect effect/total effect) ranged from 8.7 to 20.2% for hub genes from the *darkred* module and from 10.3 to 17.0% for hub genes from the *gray60* module. Significant (*P* < 0.05) mediation was observed for five of these genes: *FZD4*, *COL15A1*, *GPR124*, *COL6A1*, and *COL1A1*.

## Discussion

Using WGCNA as an alternative method to conventional differential gene expression analyses, we found interesting clusters of co-expressed genes and intramodular hub genes in placental tissue that were correlated with both maternal pre-pregnancy BMI and birth weight of the newborns. Identified gene modules were mainly related to the immune and vascular system, organ and tissue development, and extracellular structure organization. Mediation analyses suggested that identified genes may be on the causal pathway of the association between maternal BMI and newborn weight, with significant mediation effects observed for five of the hub genes (*FZD4*, *COL15A1*, *GPR124*, *COL6A1*, and *COL1A1*).

One of the modules (*darkgray*) was associated with maternal pre-pregnancy BMI and with low birth weight [below the 10^th^ percentile (2643 g)] and was enriched for genes involved in the defense response to external stimuli. In adults, excess adiposity has been linked to a reduced immune function and host defense, possibly due to the obesity-associated low-grade chronic inflammation and disturbed levels of circulating nutrients and metabolic hormones (Milner and Beck, 2012). Suboptimal fetal growth has been found to increase the risk of infectious disease mortality in childhood (Ashworth, 1998), and is associated with a reduced infant immune response to routine vaccination (Obanewa and Newell, 2017). Alterations to the hypothalamic–pituitary–adrenal axis, direct nutritional effects, and an impaired transfer of immunity from mother to child have been proposed as mechanisms by which prenatal nutritional exposures may have a lasting impact on the development of the immune system (Palmer, 2011). We also found an enrichment of genes involved in myeloid cell differentiation in the *darkgray* module, suggesting that maternal BMI might alter immune function through alterations in hematopoietic cell development. In line with this, a murine model has recently shown that a maternal high fat diet restricts the expansion and renewal of fetal hematopoietic stem cells, and promotes differentiation of both lymphoid and myeloid cell lineages (Kamimae-Lanning et al., 2015).

*AHSP*, *SLC4A1*, and *HBQ1* genes were identified as hub genes in the *darkgray* module and were positively correlated with pre-pregnancy BMI, and negatively with low birth weight. *AHSP* acts as a chaperone to prevent the harmful aggregation of alpha-hemoglobin during normal erythroid cell development. *SLC4A1* is expressed in the erythrocyte plasma membrane and functions both as a transporter that mediates electroneutral anion exchange across the cell membrane and as a structural protein. *HBQ1* is a hemoglobin gene found in human fetal erythroid tissue. Although this is the first study suggesting a role of these genes in developmental programming by maternal BMI, studies on adults have demonstrated an upregulation of these genes in association with BMI or obesity in matrices such as adipose tissue (Poitou et al., 2015), whole blood (*SLC4A1*) (Wang et al., 2017), peripheral blood (*AHSP*) (Ghosh et al., 2010), and meniscus samples (*HBQ1*) (Rai et al., 2014). In addition, *AHSP* and *SLC4A1* expression has been found to be higher in placental tissue of large-for-gestational-age infants (Ahlsson et al., 2015), whereas decreased placental *AHSP* mRNA levels were found in pregnancies complicated by low platelet syndrome, fetal death, and intrauterine growth restriction (although not significant for the latter) (Emanuelli et al., 2008).

The two other identified modules (*darkred* and *gray60*) were positively correlated with maternal BMI, maternal obesity, and birth weight. The correlation between these modules (Pearson correlation between eigengenes = 0.648) is reflected in the overlap in GO terms related to tissue and organ development. The *darkred* module contained several vascular-related genes, with blood vessel morphogenesis as top GO term, and vascular smooth muscle contraction as top KEGG pathway. Alterations in placental vasculature have an impact on the exchange of nutrients and gasses between mother and fetus, thereby affecting fetal growth. Animal models suggest an increased placental nutrient transport capacity as the mechanistic link between maternal obesity and fetal overgrowth, whereas reduced vascular branching in placentas with hypertensive disorders such as preeclampsia may be an underlying mechanism restricting fetal growth in obese pregnancies (Howell and Powell, 2017). In a RNA-sequencing study on placenta from 24 subjects, maternal obesity increased markers of inflammation and oxidative stress and decreased regulators of angiogenesis (HIF-1α and VEGF-A) (Saben et al., 2014). In a mouse model, however, maternal placental HIF-1α protein was elevated by maternal obesity (Fernandez-Twinn et al., 2017), supporting the hypothesis that obesity during pregnancy is associated with placental hypoxia, resulting in an induction of angiogenesis to enhance fetoplacental vascular growth (Desoye, 2018).

Consistent with the functional enrichment analyses, we identified hub genes related to blood vessel morphogenesis in the *darkred* module: *TBXA2R*, *FZD4*, *COL15A1*, and *TBX2* were positively associated with maternal BMI and/or obesity and with birth weight. *TBXA2R* is the receptor for Thromboxane A2 (*TXA2*), a marker of platelet activation that is greater in obese than in lean subjects (Graziani et al., 2011). In line with this is the upregulation of adipose tissue *TBXA2R* in obese mice, with genetic models suggesting a role for *TXA2* in modulating peripheral tissue insulin sensitivity and adipose tissue fibrosis (Lei et al., 2015). The role of *TBXA2R* in obese pregnancies, however, is still unclear. It has recently been suggested that an impaired vasoconstriction and vasodilatation in myometrial arteries from obese women may be linked to increased *TX2A* levels, although *TBXA2R* expression in endothelial and smooth muscle cells was not affected by maternal BMI in that study (Hayward et al., 2014). *FZD4*, a member of the frizzled gene family, encodes a protein that acts as a receptor for wingless (Wnt) proteins and plays an important role in retinal vascularization. In male adults, hyperinsulinemia was found to be associated with a decreased expression of Wnt signaling genes (including *FZD4*) in adipose tissue, while expression was increased in skeletal muscle, which might reflect a compensatory mechanism to increase muscle glucose uptake and to generate new fat cells (Karczewska-Kupczewska et al., 2016). Another hub gene related to blood vessel morphogenesis, *TBX2*, is implicated in developmental processes such as cell fate regulation, tissue and organ morphogenesis (Abrahams et al., 2010). An upregulation of this gene in association with BMI in adults has been demonstrated (Wang et al., 2017), but the importance of this gene in fetal metabolic programming needs further investigation.

Other identified hub genes in the *darkred* module were *EHD2*, *CDC42EP2*, *GPR124*, *VIM*, and *EFEMP2*. *EHD2* functions in membrane trafficking between the plasma membrane and endosomes, and has been associated with obesity in mice models (Sonne et al., 2017). *CDC42EP2* and *VIM* are involved in maintaining cell shape and integrity by stabilizing cytoskeletal interactions. VIM expression in islet cells is higher in type 2 diabetes (Roefs et al., 2017), although VIM protein levels were lower in placenta from obese pregnant women with normal glucose tolerance (Oliva et al., 2012). *GPR124* controls central nervous system angiogenesis and blood–brain barrier integrity by promoting canonical Wnt signaling via *FZD4* (Zhou and Nathans, 2014). A recent study in mice suggests that maternal obesity during pregnancy increases the permeability of the blood–brain barrier, which might affect the postnatal development of the hypothalamic circuits that regulate body weight through excessive exposure to factors such as leptin or ghrelin (Kim et al., 2016). Lastly, the *EFEMP2* gene is implicated in blood coagulation, activation of complement and determination of cell fate during development, and has been reported to be upregulated in adipose tissue of mice (Mulder et al., 2016).

In line with our findings for the *gray60* module, the PI3K-Akt signaling and focal adhesion pathways were found to be upregulated in the placenta of non-diabetic macrosomia (Song et al., 2018). Altered maternal nutrient partitioning and placental upregulation of metabolic signaling pathways, including PI3K, have also been observed in obese mice (Sferruzzi-Perri et al., 2013). Extracellular structure organization was the most enriched biological process in the *gray60* module. The extracellular matrix (ECM) plays a crucial role in adipocyte development and function. Instability of the ECM may be a direct consequence of adipocyte overgrowth, or may indirectly result from obesity-associated hypoxia (Mariman and Wang, 2010). Several studies have reported an increase in gene expression of collagens, growth factors, and enzymatic regulators of the skeletal muscle ECM in obesity (Martinez-Huenchullan et al., 2017). In line with this, maternal obesity has been found to enhance collagen accumulation and cross-linking in skeletal muscle of ovine offspring (Huang et al., 2012). Although this is the first study to report an upregulation of collagen genes in association with maternal BMI in the placenta, a thickening of the trophoblastic basement membrane with increasing amounts of collagen has been demonstrated in the context of maternal diabetes (Vambergue and Fajardy, 2011). In pre-eclamptic placentas, collagen genes have been found to be downregulated (He et al., 2015).

An advantage of our data analysis approach is that WGCNA is a method that takes into account the correlation between genes. WGCNA clusters highly co-expressed genes into modules of conserved biological function (Zhao et al., 2010). In addition, it quantifies the extent to which genes share the same neighbors, allowing the identification of highly connected genes inside each module. Because of their key position inside the network, such hub genes are likely to be biomarkers of a specific phenotype or disease status. Another strength of this study is the availability of placental microarray data from a relatively large study population that is representative for the gestational segment of the population at large (Janssen et al., 2017). In contrast to typical transcriptome studies contrasting an often limited number of adverse (e.g., obese or macrosomic) and normal phenotypes, our study results reflect normal variations in maternal and newborn traits. However, a small variation in phenotypic traits is likely to result in rather modest differences in gene expression, which may explain the absence of significant differentially expressed genes after multiple testing correction in the univariate models. Nevertheless, using a co-expression analysis, we were able to pick up dysregulated gene networks and hub genes associated with such subtle differences in maternal BMI and newborn weight. By focusing on groups of coordinately expressed genes, WGCNA has the advantage of heavily reducing the number of multiple comparisons and providing a functional interpretation that is biologically significant.

As in all epidemiological studies, findings of this study do not necessarily reflect causal associations. Observed alterations in expression of some genes may be due to confounding factors that also correlate with transcript variability. However, in contrast to most studies using WGCNA, associations between expression levels (of modules and genes) and phenotypic traits were corrected for potential confounders such as ethnicity, maternal smoking, and gestational weight gain. In addition, the placenta represents a heterogeneous mixture of cells and expression levels are expected to vary between cell types. Although biopsies were taken at a fixed location at the fetal side using a standardized sampling method, residual confounding by cell composition cannot be ruled out completely. Finally, associations observed between low birth weight and (hub genes inside) the *darkgray* module should be interpreted with caution because of the low number (*n* = 19) of observations inside the low birth weight category.

## Conclusion

Using a co-expression approach, we identified pivotal placental gene clusters that may shed new light on the molecular link between maternal and offspring metabolic health. Interestingly, this study indicated genes involved in immune defense and erythrocyte-related hub genes to be upregulated in association with maternal pre-pregnancy BMI and downregulated in association with low birth weight. Moreover, modules enriched for developmental, vascular, and extracellular matrix-related genes were positively correlated with maternal BMI and birth weight. Given the critical role of the placenta in regulating gestational development and the intrauterine environment, the identified gene networks may reflect molecular mechanisms underlying placental dysfunction associated with BMI and may be involved in fetal metabolic programming.

## Ethics Statement

This study was carried out in accordance with the recommendations of the Ethical Committee of Hasselt University and the East-Limburg Hospital with written informed consent from all subjects. All subjects gave written informed consent in accordance with the Declaration of Helsinki. The protocol was approved by the Ethical Committee of Hasselt University and the East-Limburg Hospital.

## Author Contributions

TN coordinates the ENVIR*ON*AGE birth cohort and designed the current study together with BC, MP, and KV. KN prepared the placental samples and TdK was responsible for the microarray analysis. BC performed the statistical analysis and, with contribution of MT and EW, the bioinformatical analysis. BC and MT wrote the first draft of the manuscript. All authors were involved in data interpretation and critical revision of the manuscript.

## Conflict of Interest Statement

The authors declare that the research was conducted in the absence of any commercial or financial relationships that could be construed as a potential conflict of interest.

## References

[B1] Abarca-Gómez L. Abdeen Z. A. Hamid Z. A. Abu-Rmeileh N. M. Acosta-Cazares B. Acuin C. (2017). Worldwide trends in body-mass index, underweight, overweight, and obesity from 1975 to 2016: a pooled analysis of 2416 population-based measurement studies in 128.9 million children, adolescents, and adults. *Lancet* 390 2627–2642. 10.1016/S0140-6736(17)32129-3 29029897 PMC5735219

[B2] Abrahams A. Parker M. I. Prince S. (2010). The T-box transcription factor Tbx2: its role in development and possible implication in cancer. *IUBMB Life* 62 92–102. 10.1002/iub.275 19960541

[B3] Ahlsson F. Akerud H. Schijven D. Olivier J. Sundstrom-Poromaa I. (2015). Gene expression in placentas from nondiabetic women giving birth to large for gestational age infants. *Reprod. Sci.* 22 1281–1288. 10.1177/1933719115578928 25824011

[B4] Altmäe S. Segura M. T. Esteban F. J. Bartel S. Brandi P. Irmler M. (2017). Maternal pre-pregnancy obesity is associated with altered placental transcriptome. *PLoS One* 12:e0169223. 10.1371/journal.pone.0169223 28125591 PMC5268451

[B5] Ashworth A. (1998). Effects of intrauterine growth retardation on mortality and morbidity in infants and young children. *Eur. J. Clin. Nutr.* 52(Suppl. 1), S34–S41; discussion S41–S42. fpsyg-10-00849 9511018

[B6] Aune D. Saugstad O. D. Henriksen T. Tonstad S. (2014). Maternal body mass index and the risk of fetal death, stillbirth, and infant death: a systematic review and meta-analysis. *JAMA* 311 1536–1546. 10.1001/jama.2014.2269 24737366

[B7] Burton G. J. Fowden A. L. Thornburg K. L. (2016). Placental origins of chronic disease. *Physiol. Rev.* 96 1509–1565. 10.1152/physrev.00029.2015 27604528 PMC5504455

[B8] Cox B. Martens E. Nemery B. Vangronsveld J. Nawrot T. S. (2013). Impact of a stepwise introduction of smoke-free legislation on the rate of preterm births: analysis of routinely collected birth data. *BMJ* 346:f441. 10.1136/bmj.f441 23412829 PMC3573179

[B9] Desoye G. (2018). The human placenta in diabetes and obesity: friend or foe? The 2017 Norbert Freinkel award lecture. *Diabetes Care* 41 1362–1369. 10.2337/dci17-0045 29934479

[B10] Emanuelli M. Sartini D. Rossi V. Corradetti A. Landi B. Vianna C. R. (2008). Alpha-hemoglobin-stabilizing protein (AHSP) in hemolysis, elevated liver enzyme, and low platelet (HELLP) syndrome, intrauterine growth restriction (IUGR) and fetal death. *Cell Stress Chaperones* 13 67–71. 10.1007/s12192-008-0009-5 18347943 PMC2666222

[B11] Fernandez-Twinn D. S. Gascoin G. Musial B. Carr S. Duque-Guimaraes D. Blackmore H. L. (2017). Exercise rescues obese mothers’ insulin sensitivity, placental hypoxia and male offspring insulin sensitivity. *Sci. Rep.* 7:44650. 10.1038/srep44650 28291256 PMC5349590

[B12] Gaillard R. (2015). Maternal obesity during pregnancy and cardiovascular development and disease in the offspring. *Eur. J. Epidemiol.* 30 1141–1152. 10.1007/s10654-015-0085-7 26377700 PMC4684830

[B13] Ghosh S. Dent R. Harper M. E. Gorman S. A. Stuart J. S. Mcpherson R. (2010). Gene expression profiling in whole blood identifies distinct biological pathways associated with obesity. *BMC Med. Genomics* 3:56. 10.1186/1755-8794-3-56 21122113 PMC3014865

[B14] Graziani F. Biasucci L. M. Cialdella P. Liuzzo G. Giubilato S. Della Bona R. (2011). Thromboxane production in morbidly obese subjects. *Am. J. Cardiol.* 107 1656–1661. 10.1016/j.amjcard.2011.01.053 21439532

[B15] Hayward C. E. Cowley E. J. Mills T. A. Sibley C. P. Wareing M. (2014). Maternal obesity impairs specific regulatory pathways in human myometrial arteries. *Biol. Reprod.* 90:65. 10.1095/biolreprod.113.112623 24478391

[B16] He P. Shao D. Ye M. Zhang G. (2015). Analysis of gene expression identifies candidate markers and pathways in pre-eclampsia. *J. Obstet. Gynaecol.* 35 578–584. 10.3109/01443615.2014.990430 25528892

[B17] Howell K. R. Powell T. L. (2017). Effects of maternal obesity on placental function and fetal development. *Reproduction* 153 R97–R108. 10.1530/rep-16-0495 27864335 PMC5432127

[B18] Huang Y. Zhao J. X. Yan X. Zhu M. J. Long N. M. Mccormick R. J. (2012). Maternal obesity enhances collagen accumulation and cross-linking in skeletal muscle of ovine offspring. *PLoS One* 7:e31691. 10.1371/journal.pone.0031691 22348119 PMC3279401

[B19] Janssen B. G. Madhloum N. Gyselaers W. Bijnens E. Clemente D. B. Cox B. (2017). Cohort profile: the ENVIRonmental influence ON early AGEing (ENVIRONAGE): a birth cohort study. *Int. J. Epidemiol.* 46 1386m–1387m. 10.1093/ije/dyw269 28089960

[B20] Johnson W. E. Li C. Rabinovic A. (2007). Adjusting batch effects in microarray expression data using empirical Bayes methods. *Biostatistics* 8 118–127. 10.1093/biostatistics/kxj037 16632515

[B21] Kamimae-Lanning A. N. Krasnow S. M. Goloviznina N. A. Zhu X. Roth-Carter Q. R. Levasseur P. R. (2015). Maternal high-fat diet and obesity compromise fetal hematopoiesis. *Mol. Metab.* 4 25–38. 10.1016/j.molmet.2014.11.001 25685687 PMC4314531

[B22] Karczewska-Kupczewska M. Stefanowicz M. Matulewicz N. Nikolajuk A. Straczkowski M. (2016). Wnt signaling genes in adipose tissue and skeletal muscle of humans with different degrees of insulin sensitivity. *J. Clin. Endocrinol. Metab.* 101 3079–3087. 10.1210/jc.2016-1594 27218273

[B23] Kim D. W. Glendining K. A. Grattan D. R. Jasoni C. L. (2016). Maternal obesity in the mouse compromises the blood-brain barrier in the arcuate nucleus of offspring. *Endocrinology* 157 2229–2242. 10.1210/en.2016-1014 27054554

[B24] Kim S. (2015). ppcor: an R package for a fast calculation to semi-partial correlation coefficients. *Commun. Stat. Appl. Methods* 22 665–674. 10.5351/CSAM.2015.22.6.665 26688802 PMC4681537

[B25] Kleinrouweler C. E. Van Uitert M. Moerland P. D. Ris-Stalpers C. Van Der Post J. A. Afink G. B. (2013). Differentially expressed genes in the pre-eclamptic placenta: a systematic review and meta-analysis. *PLoS One* 8:e68991. 10.1371/journal.pone.0068991 23874842 PMC3709893

[B26] Konkel L. (2016). Lasting impact of an ephemeral organ: the role of the placenta in fetal programming. *Environ. Health Perspect.* 124 A124–A129. 10.1289/ehp.124-A124 27479992 PMC4937843

[B27] Langfelder P. Horvath S. (2008). WGCNA: an R package for weighted correlation network analysis. *BMC Bioinformatics* 9:559. 10.1186/1471-2105-9-559 19114008 PMC2631488

[B28] Lei X. Li Q. Rodriguez S. Tan S. Y. Seldin M. M. Mclenithan J. C. (2015). Thromboxane synthase deficiency improves insulin action and attenuates adipose tissue fibrosis. *Am. J. Physiol. Endocrinol. Metab.* 308 E792–E804. 10.1152/ajpendo.00383.2014 25738781 PMC4420899

[B29] Madeleneau D. Buffat C. Mondon F. Grimault H. Rigourd V. Tsatsaris V. (2015). Transcriptomic analysis of human placenta in intrauterine growth restriction. *Pediatr. Res.* 77 799–807. 10.1038/pr.2015.40 25734244

[B30] Marchi J. Berg M. Dencker A. Olander E. K. Begley C. (2015). Risks associated with obesity in pregnancy, for the mother and baby: a systematic review of reviews. *Obes. Rev.* 16 621–638. 10.1111/obr.12288 26016557

[B31] Mariman E. C. M. Wang P. (2010). Adipocyte extracellular matrix composition, dynamics and role in obesity. *Cell. Mol. Life Sci.* 67 1277–1292. 10.1007/s00018-010-0263-4 20107860 PMC2839497

[B32] Martinez-Huenchullan S. Mclennan S. V. Verhoeven A. Twigg S. M. Tam C. S. (2017). The emerging role of skeletal muscle extracellular matrix remodelling in obesity and exercise. *Obes. Rev.* 18 776–790. 10.1111/obr.12548 28474421

[B33] Milner J. J. Beck M. A. (2012). The impact of obesity on the immune response to infection. *Proc. Nutr. Soc.* 71 298–306. 10.1017/s0029665112000158 22414338 PMC4791086

[B34] Mulder P. Morrison M. C. Verschuren L. Liang W. Van Bockel J. H. Kooistra T. (2016). Reduction of obesity-associated white adipose tissue inflammation by rosiglitazone is associated with reduced non-alcoholic fatty liver disease in LDLr-deficient mice. *Sci. Rep.* 6:31542. 10.1038/srep31542 27545964 PMC4992869

[B35] Nishizawa H. Ota S. Suzuki M. Kato T. Sekiya T. Kurahashi H. (2011). Comparative gene expression profiling of placentas from patients with severe pre-eclampsia and unexplained fetal growth restriction. *Reprod. Biol. Endocrinol.* 9:107. 10.1186/1477-7827-9-107 21810232 PMC3199758

[B36] Obanewa O. Newell M. L. (2017). Maternal nutritional status during pregnancy and infant immune response to routine childhood vaccinations. *Future Virol.* 12 525–536. 10.2217/fvl-2017-0021 29225661 PMC5716389

[B37] Oliva K. Barker G. Riley C. Bailey M. J. Permezel M. Rice G. E. (2012). The effect of pre-existing maternal obesity on the placental proteome: two-dimensional difference gel electrophoresis coupled with mass spectrometry. *J. Mol. Endocrinol.* 48 139–149. 10.1530/jme-11-0123 22301947

[B38] Palmer A. C. (2011). Nutritionally mediated programming of the developing immune system. *Adv. Nutr.* 2 377–395. 10.3945/an.111.000570 22332080 PMC3183589

[B39] Poitou C. Perret C. Mathieu F. Truong V. Blum Y. Durand H. (2015). Bariatric surgery induces disruption in inflammatory signaling pathways mediated by immune cells in adipose tissue: a RNA-Seq study. *PLoS One* 10:e0125718. 10.1371/journal.pone.0125718 25938420 PMC4418598

[B40] Rai M. F. Patra D. Sandell L. J. Brophy R. H. (2014). Relationship of gene expression in the injured human meniscus to body mass index: a biologic connection between obesity and osteoarthritis. *Arthritis Rheumatol.* 66 2152–2164. 10.1002/art.38643 24692131 PMC4116431

[B41] Roefs M. M. Carlotti F. Jones K. Wills H. Hamilton A. Verschoor M. (2017). Increased vimentin in human alpha- and beta-cells in type 2 diabetes. *J. Endocrinol.* 233 217–227. 10.1530/joe-16-0588 28348116

[B42] Saben J. Lindsey F. Zhong Y. Thakali K. Badger T. M. Andres A. (2014). Maternal obesity is associated with a lipotoxic placental environment. *Placenta* 35 171–177. 10.1016/j.placenta.2014.01.003 24484739 PMC3978121

[B43] Sabri A. Lai D. D’silva A. Seeho S. Kaur J. Ng C. (2014). Differential placental gene expression in term pregnancies affected by fetal growth restriction and macrosomia. *Fetal Diagn. Ther.* 36 173–180. 10.1159/000360535 24685769

[B44] Sferruzzi-Perri A. N. Vaughan O. R. Haro M. Cooper W. N. Musial B. Charalambous M. (2013). An obesogenic diet during mouse pregnancy modifies maternal nutrient partitioning and the fetal growth trajectory. *FASEB J.* 27 3928–3937. 10.1096/fj.13-234823 23825226

[B45] Sober S. Reiman M. Kikas T. Rull K. Inno R. Vaas P. (2015). Extensive shift in placental transcriptome profile in preeclampsia and placental origin of adverse pregnancy outcomes. *Sci. Rep.* 5:13336. 10.1038/srep13336 26268791 PMC4542630

[B46] Song G. Y. Na Q. Wang D. Qiao C. (2018). Microarray expression profile of lncRNAs and mRNAs in the placenta of non-diabetic macrosomia. *J. Dev. Orig. Health Dis.* 9 191–197. 10.1017/s2040174417000927 29141697

[B47] Sonne S. B. Yadav R. Yin G. Dalgaard M. D. Myrmel L. S. Gupta R. (2017). Obesity is associated with depot-specific alterations in adipocyte DNA methylation and gene expression. *Adipocyte* 6 124–133. 10.1080/21623945.2017.1320002 28481699 PMC5477735

[B48] Supek F. Bosnjak M. Skunca N. Smuc T. (2011). REVIGO summarizes and visualizes long lists of gene ontology terms. *PLoS One* 6:e21800. 10.1371/journal.pone.0021800 21789182 PMC3138752

[B49] Tingley D. Yamamoto T. Hirose K. Keele L. Imai K. (2014). Mediation: R package for causal mediation analysis. *J. Stat. Softw.* 59 1–38. 10.18637/jss.v059.i0526917999

[B50] Vambergue A. Fajardy I. (2011). Consequences of gestational and pregestational diabetes on placental function and birth weight. *World J. Diabetes* 2 196–203. 10.4239/wjd.v2.i11.196 22087356 PMC3215769

[B51] Wang J. Duncan D. Shi Z. Zhang B. (2013). WEB-based GEne SeT AnaLysis Toolkit (WebGestalt): update 2013. *Nucleic Acids Res.* 41 W77–W83. 10.1093/nar/gkt439 23703215 PMC3692109

[B52] Wang W. Jiang W. Hou L. Duan H. Wu Y. Xu C. (2017). Weighted gene co-expression network analysis of expression data of monozygotic twins identifies specific modules and hub genes related to BMI. *BMC Genomics* 18:872. 10.1186/s12864-017-4257-6 29132311 PMC5683603

[B53] Zhang B. Horvath S. (2005). A general framework for weighted gene co-expression network analysis. *Stat. Appl. Genet. Mol. Biol.* 4:Article17. 10.2202/1544-6115.1128 16646834

[B54] Zhang B. Kirov S. Snoddy J. (2005). WebGestalt: an integrated system for exploring gene sets in various biological contexts. *Nucleic Acids Res.* 33 W741–W748. 10.1093/nar/gki475 15980575 PMC1160236

[B55] Zhao W. Langfelder P. Fuller T. Dong J. Li A. Hovarth S. (2010). Weighted gene coexpression network analysis: state of the art. *J. Biopharm. Stat.* 20 281–300. 10.1080/10543400903572753 20309759

[B56] Zhou Y. Nathans J. (2014). Gpr124 controls CNS angiogenesis and blood-brain barrier integrity by promoting ligand-specific canonical wnt signaling. *Dev. Cell* 31 248–256. 10.1016/j.devcel.2014.08.018 25373781 PMC4223636

