# Supplementary material for: A Co-expression Analysis of the Placental Transcriptome in Association With Maternal Pre-pregnancy BMI and Newborn Birth Weight
Source: Front Genet. 2019 Apr 29;10:354. doi: 10.3389/fgene.2019.00354 (PMC6501552; doi:10.3389/fgene.2019.00354)
Supplement: Supplementary file 1 [file Data_Sheet_1.docx]

**Supplementary Files**

**A Co-expression Analysis of the Placental Transcriptome in Association With Maternal Pre-pregnancy BMI and Newborn Birth Weight**

Bianca Cox^1†^, Maria Tsamou^1†^, Karen Vrijens^1^, Kristof Y. Neven^1^, Ellen Winckelmans^1^, Theo M. de Kok^2^, Michelle Plusquin^1^, Tim S. Nawrot^1,3^

^†^These authors contributed equally to this work.

**Table of contents**

**Figures**

**Figure S1.** Histogram of newborn birth weight and scatterplot of newborn birth weight versus maternal pre-pregnancy BMI.

**Figure S2.** Hierarchical clustering dendrogram of the module eigengenes.

**Tables**

**Table S1.** Characteristics of the ENVIR*ON*AGE sample compared to all births in Flanders.

**Table S2.** Genes associated with both maternal pre-pregnancy BMI and birth weight in univariate models.

**Table S3.** Gene ontology terms significantly enriched in the modules of interest.

**Table S4.** KEGG pathways significantly enriched in the modules of interest.

**Table S5.** Hub genes for the modules of interest.

**Table S6.** Sensitivity analysis: exclusion of non-European babies.

**Table S7.** Sensitivity analysis: exclusion of mothers with gestational diabetes.

**Table S8.** Sensitivity analysis: exclusion of mothers with gestational hypertension.

**Table S9.** Mediation analyses.


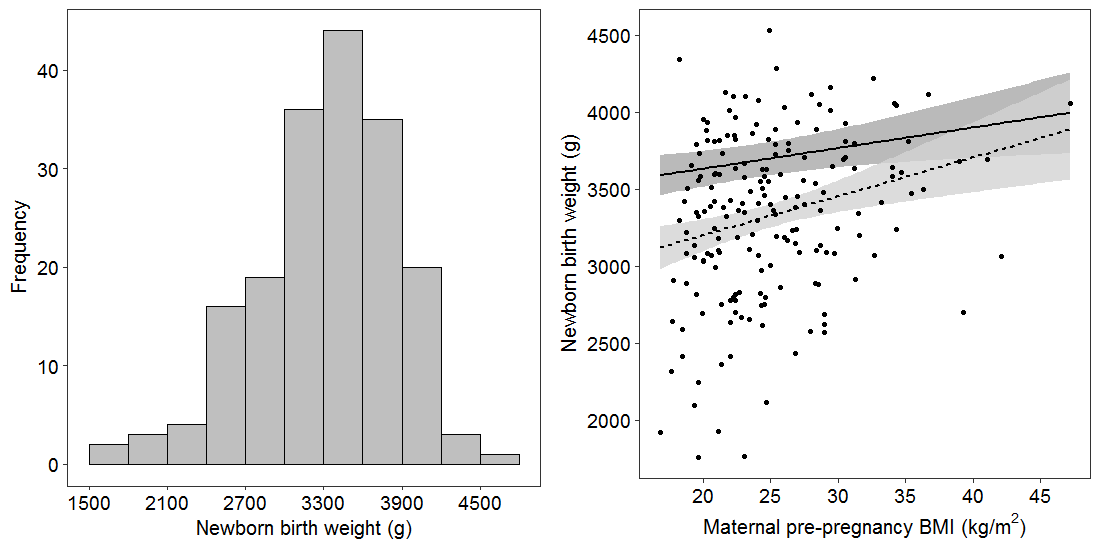


**Figure S1.** Left panel: histogram of newborn birth weight. Right panel: scatterplot of newborn birth weight versus maternal pre-pregnancy BMI with unadjusted (dashed) and adjusted (solid) regression lines together with 95% confidence intervals. The model was adjusted for date of delivery, newborn sex, gestational age, ethnicity, parity, maternal age, maternal smoking, and weight gain during pregnancy.


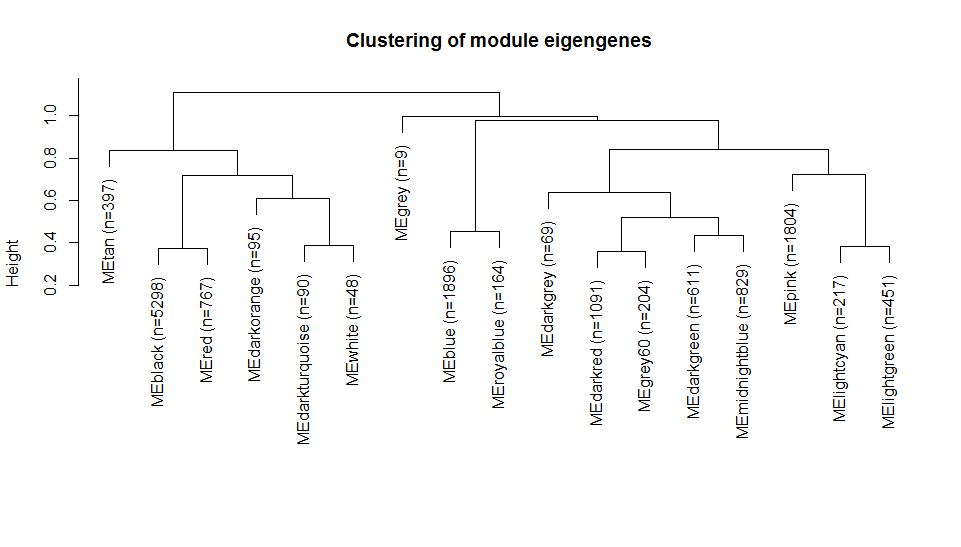
**Figure S2.** Hierarchical clustering dendrogram of the module eigengenes (MEs). Using a cut height of 0.35 (merging modules with a correlation between MEs of 0.65 or greater), we obtained 17 gene modules, identified by a color name and with the *gray* module containing unclustered genes not assigned to any module. The height of the dendrogram represents the dissimilarity of MEs, defined as one minus their correlation. The number of genes inside each module is shown between parentheses.

**Table S1.** Characteristics of the ENVIR*ON*AGE sample compared to all births in Flanders.

| **Characteristics** | ENVIR*ON*AGE (n=183) | Flanders (n=606877)^§^ |
| --- | --- | --- |
| Mother |  |  |
| Age, *years* | 29.9 (25.0-35.0) | 29.5 (23.5-35.8) |
| Parity |  |  |
| *1* | 48.6 | 46.9 |
| *2* | 38.8 | 34.7 |
| *≥3* | 12.6 | 18.4 |
|  |  |  |
| Newborn |  |  |
| Boys | 51.9 | 51.4 |
| Gestational age, *weeks* | 38.9 (36-41) | 38.9 (37-41) |
| Birth weight, *g* | 3328 (2643-3963) | 3360 (2740-3965) |
| European ethnicity | 85.8 | 87.7 |

Values are percentages or means (10^th^-90^th^ percentiles). ^§^2002-2011 (Cox et al., 2013).

**Table S2.** Genes associated^*^ with both maternal pre-pregnancy BMI and newborn birth weight in univariate models.

| **Gene symbol** | **Gene name** | ***P*_BMI_** | ***P*_BW_** |
| --- | --- | --- | --- |
| **Upregulated with maternal BMI and newborn BW** | |  |  |
| *C7* | complement component 7 | 0.00021 | 0.00102 |
| *COL8A2* | collagen type VIII alpha 2 chain | 0.00135 | 0.04001 |
| *FZD4* | frizzled class receptor 4 | 0.00147 | 0.00780 |
| *MATN2* | matrilin 2 | 0.00185 | 0.01772 |
| *SNAI2* | snail family zinc finger 2 | 0.00268 | 0.00726 |
| *ADCY3* | adenylate cyclase 3 | 0.00327 | 0.00994 |
| *CLIP3* | CAP-GLY domain containing linker protein 3 | 0.00379 | 0.00406 |
| *NFIA* | nuclear factor I/A | 0.00394 | 0.00146 |
| *GIMAP8* | GTPase, IMAP family member 8 | 0.00527 | 0.03228 |
| *ZCCHC24* | zinc finger, CCHC domain containing 24 | 0.00543 | 0.00429 |
| *RIMS3* | regulating synaptic membrane exocytosis 3 | 0.00563 | 0.01781 |
| *TENC1* | tensin like C1 domain containing phosphatase (tensin 2) | 0.00715 | 0.00632 |
| *LTBP4* | latent transforming growth factor beta binding protein 4 | 0.00755 | 0.03127 |
| *CBFA2T3* | core-binding factor, runt domain, alpha subunit 2; translocated to, 3 | 0.00766 | 0.00306 |
| *PKNOX2* | PBX/knotted 1 homeobox 2 | 0.00777 | 0.00494 |
| *PTGDS* | prostaglandin D2 synthase 21kDa (brain) | 0.00963 | 0.02962 |
| *EGFL6* | EGF-like-domain, multiple 6 | 0.01188 | 0.01204 |
| *KCNMB4* | potassium large conductance calcium-activated channel, subfamily M, beta member 4 | 0.01198 | 0.00978 |
| *NR2F1* | nuclear receptor subfamily 2, group F, member 1 | 0.01427 | 0.00010 |
| *GGT5* | gamma-glutamyltransferase 5 | 0.01461 | 0.03628 |
| *NFATC1* | nuclear factor of activated T-cells, cytoplasmic, calcineurin-dependent 1 | 0.01534 | 0.01612 |
| *COL6A1* | collagen type VI alpha 1 chain | 0.01577 | 0.00975 |
| *PRRX1* | paired related homeobox 1 | 0.01626 | 0.02647 |
| *CDC42EP2* | CDC42 effector protein (Rho GTPase binding) 2 | 0.01878 | 0.03939 |
| *CX3CR1* | chemokine (C-X3-C motif) receptor 1 | 0.01893 | 0.03535 |
| *COL15A1* | collagen type XV alpha 1 chain | 0.01914 | 0.00815 |
| *TBXA2R* | thromboxane A2 receptor | 0.01936 | 0.03422 |
| *TRPC1* | transient receptor potential cation channel subfamily C member 1 | 0.02019 | 0.04747 |
| *COL1A2* | collagen type I alpha 2 chain | 0.02235 | 0.00448 |
| *ZEB1* | zinc finger E-box binding homeobox 1 | 0.02293 | 0.01286 |
| *IFI44* | interferon-induced protein 44 | 0.02315 | 0.03817 |
| *COL16A1* | collagen type XVI alpha 1 chain | 0.02385 | 0.02786 |
| *SPON1* | spondin 1, extracellular matrix protein | 0.02451 | 0.04633 |
| *XLOC_004229* |  | 0.02471 | 0.00630 |
| *MSRB3* | methionine sulfoxide reductase B3 | 0.02517 | 0.00420 |
| *NID1* | nidogen 1 | 0.02671 | 0.00907 |
| *DIO3OS* | DIO3 opposite strand/antisense RNA (head to head) | 0.02742 | 0.00987 |
| *DEFA4* | defensin, alpha 4, corticostatin | 0.02790 | 0.02474 |
| *VSTM4* | V-set and transmembrane domain containing 4 | 0.02877 | 0.00518 |
| *COL3A1* | collagen type III alpha 1 chain | 0.02941 | 0.03494 |
| *LTBP2* | latent transforming growth factor beta binding protein 2 | 0.02983 | 0.00600 |
| *NEXN* | nexilin (F actin binding protein) | 0.03041 | 0.02693 |
| *EHD2* | EH domain containing 2 | 0.03143 | 0.02409 |
| *RUNX1T1* | RUNX1 Translocation Partner 1 | 0.03167 | 0.02026 |
| *PLA2G5* | phospholipase A2, group V | 0.03274 | 0.04921 |
| *CLEC4GP1* | C-type lectin domain family 4, member G pseudogene 1 | 0.03341 | 0.02385 |
| *KIRREL* | kin of IRRE like (Drosophila) | 0.03345 | 0.02006 |
| *AEBP1* | AE binding protein 1 | 0.03411 | 0.03380 |
| *DEFA3* | defensin, alpha 3, neutrophil-specific | 0.03420 | 0.02195 |
| *GPR124* | G-protein coupled receptor 124 | 0.03459 | 0.00424 |
| *PTPRD* | protein tyrosine phosphatase, receptor type, D | 0.03673 | 0.00968 |
| *COL1A1* | collagen type I alpha 1 chain | 0.03772 | 0.00371 |
| *MINOS1-NBL1* | MINOS1-NBL1 readthrough | 0.03940 | 0.00587 |
| *LURAP1L* | leucine rich adaptor protein 1-like | 0.04039 | 0.03966 |
| *UACA* | uveal autoantigen with coiled-coil domains and ankyrin repeats | 0.04126 | 0.01151 |
| *CAMP* | cathelicidin antimicrobial peptide | 0.04164 | 0.00565 |
| *TRIM63* | tripartite motif containing 63, E3 ubiquitin protein ligase | 0.04481 | 0.02006 |
| *CLEC11A* | C-type lectin domain family 11, member A | 0.04532 | 0.01970 |
| *CRABP1* | cellular retinoic acid binding protein 1 | 0.04574 | 0.04108 |
| **Downregulated with maternal BMI and newborn BW** | |  |  |
| *HIST3H2A* | histone cluster 3, H2a | 0.00055 | 0.00011 |
| *XLOC_008661* |  | 0.00215 | 0.00010 |
| *C10orf76* | chromosome 10 open reading frame 76 | 0.00343 | 0.00006 |
| *DDX6* | DEAD (Asp-Glu-Ala-Asp) box helicase 6 | 0.00423 | 0.00008 |
| *EFCAB2* | EF-hand calcium binding domain 2 | 0.00776 | 0.00010 |
| *NSUN4* | NOP2/Sun domain family, member 4 | 0.00999 | 0.00007 |
| *ENDOV* | endonuclease V | 0.01374 | 0.00008 |
| *RAB18* | RAB18, member RAS oncogene family | 0.01882 | 0.00005 |
| *HIST1H2AB* | histone cluster 1, H2ab | 0.02024 | 0.00007 |
| *PGAM1* | phosphoglycerate mutase 1 (brain) | 0.02144 | 0.00005 |
| *DDA1* | DET1 and DDB1 associated 1 | 0.02315 | 0.00005 |
| *INSIG2* | insulin induced gene 2 | 0.02812 | 0.00007 |
| *SNORD90* | small nucleolar RNA, C/D box 90 | 0.02820 | 0.00014 |
| *DNAJC1* | DnaJ (Hsp40) homolog, subfamily C, member 1 | 0.02924 | 0.00005 |
| *PPIL3* | peptidylprolyl isomerase (cyclophilin)-like 3 | 0.02989 | 0.00006 |
| *THAP9* | THAP domain containing 9 | 0.03107 | 0.00005 |
| *COL10A1* | collagen type X alpha 1 chain | 0.03407 | 0.00007 |
| *MCM9* | minichromosome maintenance complex component 9 | 0.03482 | 0.00009 |
| *MYOF* | Myoferlin | 0.03523 | 0.00010 |
| *SNORD43* | small nucleolar RNA, C/D box 43 | 0.03792 | 0.00011 |
| *TMEM179* | transmembrane protein 179 | 0.04050 | 0.00010 |
| *HIST2H2AA4* | histone cluster 2, H2aa4 | 0.04090 | 0.00009 |
| *ADPGK* | ADP-dependent glucokinase | 0.04381 | 0.00005 |
| *SNORD96A* | small nucleolar RNA, C/D box 96A | 0.04481 | 0.00012 |
| *ZNF101* | zinc finger protein 101 | 0.04574 | 0.00005 |
| *SNORD22* | small nucleolar RNA, C/D box 22 | 0.04667 | 0.00007 |

^*^Unadjusted *P*-values for maternal BMI (*P*_BMI_) and for newborn birth weight (*P*_BW_) < 0.05. Benjamini-Hochberg adjusted *P*-values were all > 0.05.

**Table S3.** Gene ontology (GO) terms significantly (FDR<0.05) enriched in the modules of interest.

| **GO id** | **Description** | **Effective/**  **Total size** | **FDR** |
| --- | --- | --- | --- |
| ***Darkgray* module** | |  |  |
| GO:0009620 | response to fungus | 6/26 | 2.34E-06 |
| GO:0009617 | response to bacterium | 12/298 | 9.73E-06 |
| GO:0098542 | defense response to other organism | 11/272 | 2.62E-05 |
| GO:0001906 | cell killing | 5/60 | 2.59E-03 |
| GO:0035821 | modification of morphology or physiology of other organism | 5/84 | 1.07E-02 |
| GO:0030099 | myeloid cell differentiation | 7/253 | 3.71E-02 |
| GO:0010035 | response to inorganic substance | 8/342 | 3.71E-02 |
| ***Darkred* module** | |  |  |
| GO:0048514 | blood vessel morphogenesis | 81/351 | <1.00E-16 |
| GO:0003013 | circulatory system process | 64/278 | 1.17E-11 |
| GO:0019932 | second-messenger-mediated signaling | 38/146 | 3.48E-08 |
| GO:0050673 | epithelial cell proliferation | 48/232 | 4.82E-07 |
| GO:0001655 | urogenital system development | 45/211 | 4.82E-07 |
| GO:0035239 | tube morphogenesis | 48/235 | 5.36E-07 |
| GO:0003158 | endothelium development | 26/94 | 3.24E-06 |
| GO:0007507 | heart development | 57/333 | 1.10E-05 |
| GO:1901342 | regulation of vasculature development | 35/162 | 1.10E-05 |
| GO:0007389 | pattern specification process | 42/215 | 1.14E-05 |
| GO:0071559 | response to transforming growth factor beta | 33/157 | 4.03E-05 |
| GO:0050878 | regulation of body fluid levels | 52/313 | 7.14E-05 |
| GO:0007219 | Notch signaling pathway | 26/112 | 7.53E-05 |
| GO:0001501 | skeletal system development | 51/307 | 7.79E-05 |
| GO:0007423 | sensory organ development | 50/301 | 9.26E-05 |
| GO:0018212 | peptidyl-tyrosine modification | 42/241 | 1.69E-04 |
| GO:0002521 | leukocyte differentiation | 48/292 | 1.78E-04 |
| GO:0043062 | extracellular structure organization | 39/224 | 3.43E-04 |
| GO:0045165 | cell fate commitment | 25/118 | 4.60E-04 |
| GO:0043410 | positive regulation of MAPK cascade | 51/330 | 4.60E-04 |
| GO:0061448 | connective tissue development | 30/158 | 5.14E-04 |
| GO:0030856 | regulation of epithelial cell differentiation | 19/78 | 5.14E-04 |
| GO:0033002 | muscle cell proliferation | 24/113 | 5.14E-04 |
| GO:0070371 | ERK1 and ERK2 cascade | 32/174 | 5.14E-04 |
| GO:0009187 | cyclic nucleotide metabolic process | 22/99 | 5.14E-04 |
| GO:0035637 | multicellular organismal signaling | 21/92 | 5.14E-04 |
| GO:0060485 | mesenchyme development | 30/159 | 5.14E-04 |
| GO:0007265 | Ras protein signal transduction | 39/232 | 5.14E-04 |
| GO:0001763 | morphogenesis of a branching structure | 25/124 | 7.49E-04 |
| GO:0046777 | protein autophosphorylation | 29/155 | 7.56E-04 |
| GO:0050817 | coagulation | 37/221 | 8.29E-04 |
| GO:1904019 | epithelial cell apoptotic process | 16/62 | 8.41E-04 |
| GO:0019216 | regulation of lipid metabolic process | 32/181 | 8.81E-04 |
| GO:0001503 | ossification | 40/251 | 1.17E-03 |
| GO:0001667 | ameboidal-type cell migration | 36/220 | 1.53E-03 |
| GO:0090130 | tissue migration | 28/160 | 2.83E-03 |
| GO:0043491 | protein kinase B signaling | 21/105 | 2.83E-03 |
| GO:0044236 | multicellular organism metabolic process | 17/76 | 2.85E-03 |
| GO:0040013 | negative regulation of locomotion | 30/177 | 2.86E-03 |
| GO:0001818 | negative regulation of cytokine production | 27/153 | 2.97E-03 |
| GO:0072511 | divalent inorganic cation transport | 36/229 | 3.02E-03 |
| GO:0051056 | regulation of small GTPase mediated signal transduction | 33/204 | 3.16E-03 |
| GO:0060021 | palate development | 15/64 | 3.50E-03 |
| GO:0007187 | G-protein coupled receptor signaling pathway, coupled to cyclic nucleotide second messenger | 16/71 | 3.50E-03 |
| GO:0002683 | negative regulation of immune system process | 37/241 | 3.66E-03 |
| GO:0060537 | muscle tissue development | 34/216 | 3.96E-03 |
| GO:0003012 | muscle system process | 37/243 | 4.12E-03 |
| GO:0061564 | axon development | 38/252 | 4.12E-03 |
| GO:0045444 | fat cell differentiation | 26/150 | 4.29E-03 |
| GO:1903706 | regulation of hemopoiesis | 34/218 | 4.35E-03 |
| GO:0051271 | negative regulation of cellular component movement | 28/168 | 4.79E-03 |
| GO:0007178 | transmembrane receptor protein serine/threonine kinase signaling pathway | 33/212 | 5.30E-03 |
| GO:0007517 | muscle organ development | 34/221 | 5.32E-03 |
| GO:0048568 | embryonic organ development | 39/266 | 5.37E-03 |
| GO:0048638 | regulation of developmental growth | 30/187 | 5.37E-03 |
| GO:0042692 | muscle cell differentiation | 33/213 | 5.37E-03 |
| GO:0033674 | positive regulation of kinase activity | 49/359 | 5.48E-03 |
| GO:0048732 | gland development | 40/276 | 5.48E-03 |
| GO:1903034 | regulation of response to wounding | 18/92 | 6.91E-03 |
| GO:0030099 | myeloid cell differentiation | 37/253 | 7.14E-03 |
| GO:0097485 | neuron projection guidance | 22/124 | 7.14E-03 |
| GO:0031589 | cell-substrate adhesion | 33/218 | 7.40E-03 |
| GO:0007159 | leukocyte cell-cell adhesion | 43/309 | 7.47E-03 |
| GO:0016049 | cell growth | 47/348 | 8.17E-03 |
| GO:0090287 | regulation of cellular response to growth factor stimulus | 26/160 | 9.00E-03 |
| GO:0051961 | negative regulation of nervous system development | 25/153 | 1.02E-02 |
| GO:0072507 | divalent inorganic cation homeostasis | 36/250 | 1.02E-02 |
| GO:0046434 | organophosphate catabolic process | 17/89 | 1.12E-02 |
| GO:0031279 | regulation of cyclase activity | 11/46 | 1.28E-02 |
| GO:0010463 | mesenchymal cell proliferation | 9/33 | 1.29E-02 |
| GO:0010720 | positive regulation of cell development | 39/282 | 1.32E-02 |
| GO:0035456 | response to interferon-beta | 6/16 | 1.38E-02 |
| GO:0060560 | developmental growth involved in morphogenesis | 22/132 | 1.41E-02 |
| GO:0051339 | regulation of lyase activity | 11/47 | 1.45E-02 |
| GO:0042493 | response to drug | 36/257 | 1.51E-02 |
| GO:0010975 | regulation of neuron projection development | 36/259 | 1.71E-02 |
| GO:0006875 | cellular metal ion homeostasis | 40/297 | 1.75E-02 |
| GO:0045927 | positive regulation of growth | 25/161 | 1.81E-02 |
| GO:0097503 | sialylation | 5/12 | 1.88E-02 |
| GO:0061053 | somite development | 11/49 | 1.92E-02 |
| GO:0003014 | renal system process | 13/64 | 2.03E-02 |
| GO:0006140 | regulation of nucleotide metabolic process | 20/120 | 2.04E-02 |
| GO:0032102 | negative regulation of response to external stimulus | 24/156 | 2.35E-02 |
| GO:0010644 | cell communication by electrical coupling | 6/18 | 2.35E-02 |
| GO:0023061 | signal release | 34/247 | 2.35E-02 |
| GO:0048771 | tissue remodeling | 17/97 | 2.35E-02 |
| GO:0050865 | regulation of cell activation | 42/324 | 2.50E-02 |
| GO:0071875 | adrenergic receptor signaling pathway | 5/13 | 2.55E-02 |
| GO:0031214 | biomineral tissue development | 15/82 | 2.59E-02 |
| GO:0051272 | positive regulation of cellular component movement | 38/287 | 2.61E-02 |
| GO:0040017 | positive regulation of locomotion | 39/297 | 2.64E-02 |
| GO:0042113 | B cell activation | 22/141 | 2.64E-02 |
| GO:0033500 | carbohydrate homeostasis | 21/133 | 2.77E-02 |
| GO:0001945 | lymph vessel development | 6/19 | 2.86E-02 |
| GO:0048863 | stem cell differentiation | 18/108 | 2.86E-02 |
| GO:0045926 | negative regulation of growth | 24/160 | 2.86E-02 |
| GO:0090066 | regulation of anatomical structure size | 40/309 | 2.86E-02 |
| GO:0048017 | inositol lipid-mediated signaling | 21/135 | 3.15E-02 |
| GO:0035265 | organ growth | 16/94 | 3.63E-02 |
| GO:0060840 | artery development | 11/55 | 3.97E-02 |
| GO:0010817 | regulation of hormone levels | 36/279 | 4.44E-02 |
| GO:0031128 | developmental induction | 5/15 | 4.44E-02 |
| GO:0043627 | response to estrogen | 11/56 | 4.44E-02 |
| GO:0050900 | leukocyte migration | 33/252 | 4.83E-02 |
| GO:0030900 | forebrain development | 28/205 | 4.86E-02 |
| GO:0042537 | benzene-containing compound metabolic process | 4/10 | 4.86E-02 |
| GO:0051962 | positive regulation of nervous system development | 35/272 | 4.89E-02 |
| GO:0010171 | body morphogenesis | 9/42 | 4.89E-02 |
| GO:1901654 | response to ketone | 18/115 | 4.89E-02 |
| GO:0030705 | cytoskeleton-dependent intracellular transport | 16/98 | 4.92E-02 |
| ***Gray60* module** | |  |  |
| GO:0043062 | extracellular structure organization | 27/224 | 8.18E-12 |
| GO:0001501 | skeletal system development | 27/307 | 8.45E-09 |
| GO:0007517 | muscle organ development | 21/221 | 2.81E-07 |
| GO:0060485 | mesenchyme development | 17/159 | 1.52E-06 |
| GO:0060537 | muscle tissue development | 19/216 | 3.94E-06 |
| GO:0003012 | muscle system process | 20/243 | 4.25E-06 |
| GO:0044236 | multicellular organism metabolic process | 11/76 | 1.90E-05 |
| GO:0001503 | ossification | 19/251 | 2.70E-05 |
| GO:0048568 | embryonic organ development | 19/266 | 5.86E-05 |
| GO:0048514 | blood vessel morphogenesis | 22/351 | 6.24E-05 |
| GO:0007507 | heart development | 21/333 | 9.33E-05 |
| GO:0061448 | connective tissue development | 14/158 | 1.13E-04 |
| GO:0042692 | muscle cell differentiation | 16/213 | 1.68E-04 |
| GO:0060541 | respiratory system development | 12/133 | 4.51E-04 |
| GO:0035239 | tube morphogenesis | 16/235 | 5.15E-04 |
| GO:0042476 | odontogenesis | 9/77 | 6.36E-04 |
| GO:0007389 | pattern specification process | 15/215 | 6.36E-04 |
| GO:0030323 | respiratory tube development | 11/119 | 6.36E-04 |
| GO:0051962 | positive regulation of nervous system development | 17/272 | 6.36E-04 |
| GO:0007423 | sensory organ development | 18/301 | 6.36E-04 |
| GO:2000027 | regulation of organ morphogenesis | 13/174 | 9.75E-04 |
| GO:0055123 | digestive system development | 9/84 | 1.01E-03 |
| GO:1905330 | regulation of morphogenesis of an epithelium | 11/128 | 1.01E-03 |
| GO:0010463 | mesenchymal cell proliferation | 6/33 | 1.01E-03 |
| GO:0035265 | organ growth | 9/94 | 2.20E-03 |
| GO:0048645 | animal organ formation | 6/39 | 2.50E-03 |
| GO:0051272 | positive regulation of cellular component movement | 16/287 | 3.18E-03 |
| GO:0048736 | appendage development | 9/100 | 3.18E-03 |
| GO:0001763 | morphogenesis of a branching structure | 10/124 | 3.25E-03 |
| GO:0031214 | biomineral tissue development | 8/82 | 4.07E-03 |
| GO:0040017 | positive regulation of locomotion | 16/297 | 4.22E-03 |
| GO:0007548 | sex differentiation | 11/155 | 4.26E-03 |
| GO:0048863 | stem cell differentiation | 9/108 | 4.87E-03 |
| GO:0050817 | coagulation | 13/221 | 6.84E-03 |
| GO:0035270 | endocrine system development | 7/71 | 8.44E-03 |
| GO:0007178 | transmembrane receptor protein serine/threonine kinase signaling pathway | 12/212 | 1.52E-02 |
| GO:0060231 | mesenchymal to epithelial transition | 3/10 | 1.52E-02 |
| GO:1902742 | apoptotic process involved in development | 4/23 | 1.70E-02 |
| GO:0061458 | reproductive system development | 14/280 | 1.80E-02 |
| GO:0010720 | positive regulation of cell development | 14/282 | 1.85E-02 |
| GO:0001667 | ameboidal-type cell migration | 12/220 | 1.85E-02 |
| GO:0031016 | pancreas development | 5/41 | 1.85E-02 |
| GO:1904888 | cranial skeletal system development | 5/42 | 2.02E-02 |
| GO:0072376 | protein activation cascade | 5/43 | 2.20E-02 |
| GO:1903034 | regulation of response to wounding | 7/92 | 3.04E-02 |
| GO:0045165 | cell fate commitment | 8/118 | 3.04E-02 |
| GO:0007224 | smoothened signaling pathway | 6/70 | 3.41E-02 |
| GO:0016331 | morphogenesis of embryonic epithelium | 7/95 | 3.49E-02 |
| GO:0001655 | urogenital system development | 11/211 | 3.62E-02 |
| GO:0048732 | gland development | 13/276 | 3.62E-02 |
| GO:0031128 | developmental induction | 3/15 | 3.85E-02 |
| GO:0050878 | regulation of body fluid levels | 14/313 | 3.85E-02 |
| GO:0071559 | response to transforming growth factor beta | 9/157 | 4.40E-02 |
| GO:0060571 | morphogenesis of an epithelial fold | 3/16 | 4.40E-02 |
| GO:0061564 | axon development | 12/252 | 4.40E-02 |
| GO:0090130 | tissue migration | 9/160 | 4.75E-02 |

**Table S4.** KEGG pathways significantly (FDR<0.05) enriched in the modules of interest.

| **KEGG id** | **Description** | **Effective/**  **Total size** | **FDR** |  |
| --- | --- | --- | --- | --- |
| ***Darkred* module** | |  |  |  |
| hsa04270 | Vascular smooth muscle contraction - Homo sapiens (human) | 22/79 | 1.42E-04 |  |
| hsa04022 | cGMP-PKG signaling pathway - Homo sapiens (human) | 28/124 | 2.26E-04 |  |
| hsa04924 | Renin secretion - Homo sapiens (human) | 13/42 | 3.19E-03 |  |
| hsa04713 | Circadian entrainment - Homo sapiens (human) | 14/52 | 6.68E-03 |  |
| hsa05200 | Pathways in cancer - Homo sapiens (human) | 44/289 | 7.10E-03 |  |
| hsa04724 | Glutamatergic synapse - Homo sapiens (human) | 14/56 | 9.00E-03 |  |
| hsa04020 | Calcium signaling pathway - Homo sapiens (human) | 19/91 | 9.00E-03 |  |
| hsa04921 | Oxytocin signaling pathway - Homo sapiens (human) | 20/99 | 9.00E-03 |  |
| hsa04015 | Rap1 signaling pathway - Homo sapiens (human) | 26/146 | 9.00E-03 |  |
| hsa04014 | Ras signaling pathway - Homo sapiens (human) | 27/155 | 9.01E-03 |  |
| hsa04925 | Aldosterone synthesis and secretion - Homo sapiens (human) | 13/52 | 9.65E-03 |  |
| hsa05224 | Breast cancer - Homo sapiens (human) | 19/97 | 1.33E-02 |  |
| hsa04072 | Phospholipase D signaling pathway - Homo sapiens (human) | 18/91 | 1.51E-02 |  |
| hsa04913 | Ovarian steroidogenesis - Homo sapiens (human) | 9/32 | 2.47E-02 |  |
| hsa04970 | Salivary secretion - Homo sapiens (human) | 12/52 | 2.55E-02 |  |
| hsa04611 | Platelet activation - Homo sapiens (human) | 17/89 | 2.58E-02 |  |
| hsa04972 | Pancreatic secretion - Homo sapiens (human) | 11/49 | 4.33E-02 |  |
| hsa05202 | Transcriptional misregulation in cancer - Homo sapiens (human) | 22/135 | 4.33E-02 |  |
| hsa04923 | Regulation of lipolysis in adipocytes - Homo sapiens (human) | 9/36 | 4.46E-02 |  |
| hsa04723 | Retrograde endocannabinoid signaling - Homo sapiens (human) | 11/50 | 4.52E-02 |  |
| hsa04151 | PI3K-Akt signaling pathway - Homo sapiens (human) | 33/235 | 4.66E-02 |  |
| hsa01522 | Endocrine resistance - Homo sapiens (human) | 14/73 | 4.66E-02 |  |
| ***Gray60* module** | |  |  |  |
| hsa04151 | PI3K-Akt signaling pathway - Homo sapiens (human) | 14/235 | 7.94E-03 |  |
| hsa04510 | Focal adhesion - Homo sapiens (human) | 11/152 | 7.94E-03 |  |
| hsa04974 | Protein digestion and absorption - Homo sapiens (human) | 6/50 | 1.96E-02 |  |
| hsa05205 | Proteoglycans in cancer - Homo sapiens (human) | 10/153 | 2.07E-02 |  |
| hsa04512 | ECM-receptor interaction - Homo sapiens (human) | 6/56 | 2.21E-02 |  |
| hsa04360 | Axon guidance - Homo sapiens (human) | 8/113 | 3.05E-02 |  |
| hsa04610 | Complement and coagulation cascades - Homo sapiens (human) | 5/42 | 3.05E-02 |  |

**Table S5.** Hub genes for the modules of interest, defined as |MM| ≥ 0.8 and a significant partial correlation with at least one maternal BMI variable and at least one birth weight variable.

|  |  | **Partial correlation (*P*-value)** | | | | | | | |
| --- | --- | --- | --- | --- | --- | --- | --- | --- | --- |
|  |  | **Maternal pre-pregnancy** | | | |  | **Newborn** | | |
| **Gene symbol** | **MM** | **BMI** | **Underweight** | **Overweight** | **Obese** |  | **BW** | **Low BW** | **High BW** |
| ***Darkgray* module** | |  |  |  |  |  |  |  |  |
| *XLOC_000346* | 0.85 | 0.17 (0.026) | -0.02 (0.805) | 0.02 (0.796) | 0.16 (0.036) |  | 0.04 (0.615) | -0.16 (0.040) | 0.02 (0.812) |
| *AHSP* | 0.88 | 0.17 (0.029) | -0.05 (0.541) | 0.00 (0.987) | 0.12 (0.120) |  | 0.04 (0.618) | -0.18 (0.020) | -0.02 (0.768) |
| *XLOC_013489* | 0.92 | 0.17 (0.030) | -0.01 (0.891) | 0.01 (0.866) | 0.14 (0.062) |  | 0.02 (0.799) | -0.18 (0.015) | -0.05 (0.493) |
| *SLC4A1* | 0.91 | 0.15 (0.042) | -0.04 (0.648) | -0.01 (0.883) | 0.12 (0.124) |  | 0.07 (0.356) | -0.19 (0.012) | -0.04 (0.583) |
| *HBQ1* | 0.92 | 0.15 (0.045) | -0.02 (0.783) | -0.04 (0.579) | 0.14 (0.072) |  | 0.02 (0.805) | -0.20 (0.007) | -0.02 (0.804) |
| ***Darkred* module** | |  |  |  |  |  |  |  |  |
| *FZD4* | 0.83 | 0.24 (0.001) | -0.03 (0.725) | 0.10 (0.191) | 0.25 (0.001) |  | 0.20 (0.008) | -0.06 (0.451) | 0.05 (0.532) |
| *CDC42EP2* | 0.84 | 0.18 (0.019) | -0.10 (0.172) | 0.07 (0.368) | 0.15 (0.054) |  | 0.16 (0.039) | -0.12 (0.127) | 0.02 (0.776) |
| *COL15A1* | 0.84 | 0.18 (0.019) | -0.03 (0.678) | 0.08 (0.321) | 0.17 (0.027) |  | 0.20 (0.008) | -0.08 (0.301) | 0.00 (0.985) |
| *TBXA2R* | 0.81 | 0.18 (0.019) | -0.05 (0.545) | 0.12 (0.112) | 0.13 (0.091) |  | 0.16 (0.034) | -0.02 (0.765) | 0.04 (0.568) |
| *EHD2* | 0.89 | 0.16 (0.031) | -0.04 (0.560) | 0.04 (0.622) | 0.18 (0.019) |  | 0.17 (0.024) | -0.03 (0.659) | 0.03 (0.707) |
| *GPR124* | 0.89 | 0.16 (0.035) | 0.00 (0.977) | 0.05 (0.515) | 0.18 (0.018) |  | 0.22 (0.004) | -0.05 (0.516) | 0.04 (0.612) |
| *VIM* | 0.90 | 0.15 (0.044) | -0.03 (0.706) | 0.12 (0.109) | 0.16 (0.040) |  | 0.17 (0.025) | -0.05 (0.552) | 0.04 (0.583) |
| *EFEMP2* | 0.88 | 0.15 (0.048) | -0.03 (0.697) | 0.11 (0.141) | 0.13 (0.095) |  | 0.16 (0.038) | -0.06 (0.469) | 0.04 (0.595) |
| *TBX2* | 0.83 | 0.12 (0.120) | 0.02 (0.808) | 0.07 (0.362) | 0.19 (0.015) |  | 0.18 (0.020) | -0.07 (0.380) | 0.03 (0.731) |
| ***Gray60* module** |  |  |  |  |  |  |  |  |  |
| *COL8A2* | 0.88 | 0.24 (0.001) | -0.11 (0.146) | 0.04 (0.563) | 0.21 (0.005) |  | 0.16 (0.040) | 0.01 (0.858) | 0.02 (0.807) |
| *MATN2* | 0.86 | 0.24 (0.002) | -0.09 (0.217) | 0.10 (0.176) | 0.19 (0.013) |  | 0.18 (0.018) | -0.09 (0.225) | -0.01 (0.871) |
| *KANK2* | 0.84 | 0.19 (0.011) | -0.10 (0.193) | 0.04 (0.629) | 0.18 (0.022) |  | 0.17 (0.023) | -0.05 (0.505) | 0.01 (0.860) |
| *COL6A1* | 0.83 | 0.18 (0.016) | -0.07 (0.375) | 0.06 (0.411) | 0.18 (0.016) |  | 0.20 (0.010) | -0.07 (0.387) | 0.04 (0.613) |
| *TRPC1* | 0.81 | 0.18 (0.020) | -0.19 (0.012) | 0.14 (0.066) | 0.12 (0.120) |  | 0.15 (0.047) | -0.12 (0.113) | -0.01 (0.850) |
| *COL16A1* | 0.83 | 0.17 (0.024) | -0.18 (0.017) | -0.02 (0.799) | 0.13 (0.093) |  | 0.17 (0.028) | -0.05 (0.504) | -0.04 (0.581) |
| *COL3A1* | 0.83 | 0.17 (0.029) | -0.12 (0.118) | 0.06 (0.403) | 0.13 (0.095) |  | 0.16 (0.035) | -0.13 (0.080) | -0.05 (0.544) |
| *RUNX1T1* | 0.86 | 0.16 (0.032) | -0.02 (0.839) | 0.07 (0.337) | 0.20 (0.009) |  | 0.18 (0.020) | -0.05 (0.540) | -0.03 (0.689) |
| *AEBP1* | 0.84 | 0.16 (0.034) | -0.08 (0.318) | 0.03 (0.741) | 0.18 (0.021) |  | 0.16 (0.034) | -0.08 (0.268) | -0.08 (0.269) |
| *COL1A1* | 0.82 | 0.16 (0.038) | -0.09 (0.261) | 0.08 (0.271) | 0.12 (0.124) |  | 0.22 (0.004) | -0.07 (0.366) | 0.07 (0.361) |
| *RGS11* | 0.81 | 0.13 (0.086) | -0.08 (0.310) | 0.05 (0.558) | 0.15 (0.049) |  | 0.24 (0.002) | -0.08 (0.293) | 0.00 (1.000) |

MM: module membership; BW: birth weight

**Table S6.** MM of hub genes and partial correlations of MEs and hub genes with maternal BMI and newborn birth weight variables before (n = 183) and after (n = 157) excluding non-European babies from the analysis.

| **Module eigengene/**  **Gene symbol** | **Main analysis** | | | | | | | | |  | **Excluding non-European babies** | | | | | | | | |
| --- | --- | --- | --- | --- | --- | --- | --- | --- | --- | --- | --- | --- | --- | --- | --- | --- | --- | --- | --- |
|  |  |  | **Maternal pre-pregnancy** | | | |  | **Newborn** | |  |  |  | **Maternal pre-pregnancy** | | | |  | **Newborn** | |
|  | **MM** |  | **BMI** | **Under-weight** | **Over-weight** | **Obese** |  | **BW** | **Low BW** |  | **MM** |  | **BMI** | **Under-weight** | **Over-weight** | **Obese** |  | **BW** | **Low BW** |
| ***Darkgray* module** | | | | | | | | | | | | | | | | | | | |
| **ME** |  |  | **0.15**** | **-0.05** | **-0.01** | **0.12** |  | **0.05** | **-0.19**** |  |  |  | **0.14*** | **-0.07** | **-0.03** | **0.12** |  | **0.04** | **-0.24***** |
| *XLOC_000346* | 0.85 |  | 0.17** | -0.02 | 0.02 | 0.16** |  | 0.04 | -0.16** |  | 0.84 |  | 0.16* | -0.03 | -0.01 | 0.18** |  | 0.05 | -0.20** |
| *AHSP* | 0.88 |  | 0.17** | -0.05 | 0.00 | 0.12 |  | 0.04 | -0.18** |  | 0.87 |  | 0.18** | -0.07 | -0.04 | 0.14* |  | 0.02 | -0.22*** |
| *XLOC_013489* | 0.92 |  | 0.17** | -0.01 | 0.01 | 0.14* |  | 0.02 | -0.18** |  | 0.92 |  | 0.17** | -0.03 | -0.01 | 0.17** |  | 0.03 | -0.23*** |
| *SLC4A1* | 0.91 |  | 0.15** | -0.04 | -0.01 | 0.12 |  | 0.07 | -0.19** |  | 0.90 |  | 0.16** | -0.05 | -0.04 | 0.15* |  | 0.06 | -0.24*** |
| *HBQ1* | 0.92 |  | 0.15** | -0.02 | -0.04 | 0.14* |  | 0.02 | -0.20*** |  | 0.90 |  | 0.14* | -0.04 | -0.07 | 0.15* |  | 0.02 | -0.25*** |
| ***Darkred* module** | | | | | | | | | | | | | | | | | | | |
| **ME** |  |  | **0.14*** | **-0.03** | **0.05** | **0.15*** |  | **0.13*** | **-0.03** |  |  |  | **0.13** | **-0.04** | **0.08** | **0.13** |  | **0.12** | **-0.04** |
| *FZD4* | 0.83 |  | 0.24*** | -0.03 | 0.10 | 0.25*** |  | 0.20*** | -0.06 |  | 0.83 |  | 0.27*** | -0.02 | 0.10 | 0.27*** |  | 0.18** | -0.06 |
| *CDC42EP2* | 0.84 |  | 0.18** | -0.10 | 0.07 | 0.15* |  | 0.16** | -0.12 |  | 0.83 |  | 0.17** | -0.12 | 0.07 | 0.13 |  | 0.14* | -0.14* |
| *COL15A1* | 0.84 |  | 0.18** | -0.03 | 0.08 | 0.17** |  | 0.20*** | -0.08 |  | 0.84 |  | 0.19** | -0.04 | 0.11 | 0.16* |  | 0.21*** | -0.10 |
| *TBXA2R* | 0.81 |  | 0.18** | -0.05 | 0.12 | 0.13* |  | 0.16** | -0.02 |  | 0.79 |  | 0.20** | -0.05 | 0.14* | 0.14 |  | 0.17** | -0.04 |
| *EHD2* | 0.89 |  | 0.16** | -0.04 | 0.04 | 0.18** |  | 0.17** | -0.03 |  | 0.88 |  | 0.16* | -0.05 | 0.07 | 0.16* |  | 0.17** | -0.05 |
| *GPR124* | 0.89 |  | 0.16** | 0.00 | 0.05 | 0.18** |  | 0.22*** | -0.05 |  | 0.88 |  | 0.15* | 0.00 | 0.09 | 0.15* |  | 0.23*** | -0.07 |
| *VIM* | 0.90 |  | 0.15** | -0.03 | 0.12 | 0.16** |  | 0.17** | -0.05 |  | 0.91 |  | 0.16* | -0.03 | 0.17** | 0.15* |  | 0.18** | -0.06 |
| *EFEMP2* | 0.88 |  | 0.15** | -0.03 | 0.11 | 0.13* |  | 0.16** | -0.06 |  | 0.87 |  | 0.18** | -0.03 | 0.17** | 0.13 |  | 0.19** | -0.08 |
| *TBX2* | 0.83 |  | 0.12 | 0.02 | 0.07 | 0.19** |  | 0.18** | -0.07 |  | 0.84 |  | 0.11 | 0.03 | 0.10 | 0.15* |  | 0.19** | -0.09 |
| ***Gray60* module** | | | | | | | | | | | | | | | | | | | |
| **ME** |  |  | **0.20***** | **-0.10** | **0.07** | **0.18**** |  | **0.14*** | **-0.04** |  |  |  | **0.21**** | **-0.11** | **0.13** | **0.17**** |  | **0.13** | **-0.06** |
| *COL8A2* | 0.88 |  | 0.24*** | -0.11 | 0.04 | 0.21*** |  | 0.16** | 0.01 |  | 0.88 |  | 0.24*** | -0.13 | 0.08 | 0.19** |  | 0.13 | 0.01 |
| *MATN2* | 0.86 |  | 0.24*** | -0.09 | 0.10 | 0.19** |  | 0.18** | -0.09 |  | 0.86 |  | 0.26*** | -0.11 | 0.15* | 0.18** |  | 0.19** | -0.11 |
| *KANK2* | 0.84 |  | 0.19** | -0.10 | 0.04 | 0.18** |  | 0.17** | -0.05 |  | 0.83 |  | 0.19** | -0.11 | 0.11 | 0.14* |  | 0.17** | -0.07 |
| *COL6A1* | 0.83 |  | 0.18** | -0.07 | 0.06 | 0.18** |  | 0.20*** | -0.07 |  | 0.82 |  | 0.19** | -0.08 | 0.11 | 0.16* |  | 0.21*** | -0.09 |
| *TRPC1* | 0.81 |  | 0.18** | -0.19** | 0.14* | 0.12 |  | 0.15** | -0.12 |  | 0.82 |  | 0.19** | -0.21** | 0.15* | 0.12 |  | 0.13 | -0.14* |
| *COL16A1* | 0.83 |  | 0.17** | -0.18** | -0.02 | 0.13* |  | 0.17** | -0.05 |  | 0.84 |  | 0.16* | -0.20** | 0.03 | 0.09 |  | 0.15* | -0.08 |
| *COL3A1* | 0.83 |  | 0.17** | -0.12 | 0.06 | 0.13* |  | 0.16** | -0.13* |  | 0.83 |  | 0.16** | -0.13 | 0.14* | 0.09 |  | 0.16* | -0.17** |
| *RUNX1T1* | 0.86 |  | 0.16** | -0.02 | 0.07 | 0.20*** |  | 0.18** | -0.05 |  | 0.86 |  | 0.16* | -0.01 | 0.16* | 0.16** |  | 0.18** | -0.07 |
| *AEBP1* | 0.84 |  | 0.16** | -0.08 | 0.03 | 0.18** |  | 0.16** | -0.08 |  | 0.84 |  | 0.20** | -0.09 | 0.11 | 0.19** |  | 0.18** | -0.10 |
| *COL1A1* | 0.82 |  | 0.16** | -0.09 | 0.08 | 0.12 |  | 0.22*** | -0.07 |  | 0.80 |  | 0.15* | -0.11 | 0.15* | 0.08 |  | 0.22*** | -0.10 |
| *RGS11* | 0.81 |  | 0.13* | -0.08 | 0.05 | 0.15** |  | 0.24*** | -0.08 |  | 0.78 |  | 0.11 | -0.09 | 0.10 | 0.10 |  | 0.24*** | -0.10 |

* *P*-value < 0.1; ** *P*-value < 0.05; *** *P*-value < 0.01; MM: module membership; BW: birth weight; ME: module eigengene

**Table S7.** MM of hub genes and partial correlations of MEs and hub genes with maternal BMI and newborn birth weight variables before (n = 183) and after (n = 177) excluding mothers with gestational diabetes from the analysis.

| **Module eigengene/**  **Gene symbol** | **Main analysis** | | | | | | | | |  | **Excluding mothers with gestational diabetes** | | | | | | | | |
| --- | --- | --- | --- | --- | --- | --- | --- | --- | --- | --- | --- | --- | --- | --- | --- | --- | --- | --- | --- |
|  |  |  | **Maternal pre-pregnancy** | | | |  | **Newborn** | |  |  |  | **Maternal pre-pregnancy** | | | |  | **Newborn** | |
|  | **MM** |  | **BMI** | **Under-weight** | **Over-weight** | **Obese** |  | **BW** | **Low BW** |  | **MM** |  | **BMI** | **Under-weight** | **Over-weight** | **Obese** |  | **BW** | **Low BW** |
| ***Darkgray* module** | | | | | | | | | | | | | | | | | | | |
| **ME** |  |  | **0.15**** | **-0.05** | **-0.01** | **0.12** |  | **0.05** | **-0.19**** |  |  |  | **0.16**** | **-0.05** | **0.01** | **0.12** |  | **0.05** | **-0.20**** |
| *XLOC_000346* | 0.85 |  | 0.17** | -0.02 | 0.02 | 0.16** |  | 0.04 | -0.16** |  | 0.85 |  | 0.18** | -0.02 | 0.02 | 0.17** |  | 0.03 | -0.16** |
| *AHSP* | 0.88 |  | 0.17** | -0.05 | 0.00 | 0.12 |  | 0.04 | -0.18** |  | 0.88 |  | 0.17** | -0.06 | 0.02 | 0.12 |  | 0.03 | -0.18** |
| *XLOC_013489* | 0.92 |  | 0.17** | -0.01 | 0.01 | 0.14* |  | 0.02 | -0.18** |  | 0.92 |  | 0.17** | -0.01 | 0.03 | 0.14* |  | 0.02 | -0.19** |
| *SLC4A1* | 0.91 |  | 0.15** | -0.04 | -0.01 | 0.12 |  | 0.07 | -0.19** |  | 0.91 |  | 0.16** | -0.04 | 0.01 | 0.12 |  | 0.07 | -0.21*** |
| *HBQ1* | 0.92 |  | 0.15** | -0.02 | -0.04 | 0.14* |  | 0.02 | -0.20*** |  | 0.92 |  | 0.16** | -0.02 | -0.03 | 0.14* |  | 0.02 | -0.22*** |
| ***Darkred* module** | | | | | | | | | | | | | | | | | | | |
| **ME** |  |  | **0.14*** | **-0.03** | **0.05** | **0.15*** |  | **0.13*** | **-0.03** |  |  |  | **0.12** | **-0.05** | **0.05** | **0.13*** |  | **0.11** | **0.00** |
| *FZD4* | 0.83 |  | 0.24*** | -0.03 | 0.10 | 0.25*** |  | 0.20*** | -0.06 |  | 0.83 |  | 0.22*** | -0.04 | 0.10 | 0.23*** |  | 0.20*** | -0.05 |
| *CDC42EP2* | 0.84 |  | 0.18** | -0.10 | 0.07 | 0.15* |  | 0.16** | -0.12 |  | 0.84 |  | 0.17** | -0.12 | 0.07 | 0.14* |  | 0.14* | -0.10 |
| *COL15A1* | 0.84 |  | 0.18** | -0.03 | 0.08 | 0.17** |  | 0.20*** | -0.08 |  | 0.84 |  | 0.17** | -0.05 | 0.07 | 0.16** |  | 0.19** | -0.06 |
| *TBXA2R* | 0.81 |  | 0.18** | -0.05 | 0.12 | 0.13* |  | 0.16** | -0.02 |  | 0.81 |  | 0.17** | -0.06 | 0.12 | 0.12 |  | 0.15* | -0.01 |
| *EHD2* | 0.89 |  | 0.16** | -0.04 | 0.04 | 0.18** |  | 0.17** | -0.03 |  | 0.89 |  | 0.16** | -0.06 | 0.03 | 0.17** |  | 0.16** | -0.02 |
| *GPR124* | 0.89 |  | 0.16** | 0.00 | 0.05 | 0.18** |  | 0.22*** | -0.05 |  | 0.88 |  | 0.15* | -0.01 | 0.04 | 0.17** |  | 0.21*** | -0.02 |
| *VIM* | 0.90 |  | 0.15** | -0.03 | 0.12 | 0.16** |  | 0.17** | -0.05 |  | 0.90 |  | 0.14* | -0.04 | 0.12 | 0.14* |  | 0.16** | -0.03 |
| *EFEMP2* | 0.88 |  | 0.15** | -0.03 | 0.11 | 0.13* |  | 0.16** | -0.06 |  | 0.88 |  | 0.14* | -0.04 | 0.11 | 0.11 |  | 0.15* | -0.04 |
| *TBX2* | 0.83 |  | 0.12 | 0.02 | 0.07 | 0.19** |  | 0.18** | -0.07 |  | 0.84 |  | 0.11 | 0.01 | 0.07 | 0.17** |  | 0.18** | -0.06 |
| ***Gray60* module** | | | | | | | | | | | | | | | | | | | |
| **ME** |  |  | **0.20***** | **-0.10** | **0.07** | **0.18**** |  | **0.14*** | **-0.04** |  |  |  | **0.20**** | **-0.12** | **0.07** | **0.17**** |  | **0.12** | **-0.02** |
| *COL8A2* | 0.88 |  | 0.24*** | -0.11 | 0.04 | 0.21*** |  | 0.16** | 0.01 |  | 0.88 |  | 0.24*** | -0.13 | 0.04 | 0.21*** |  | 0.14* | 0.04 |
| *MATN2* | 0.86 |  | 0.24*** | -0.09 | 0.10 | 0.19** |  | 0.18** | -0.09 |  | 0.86 |  | 0.23*** | -0.11 | 0.11 | 0.18** |  | 0.17** | -0.08 |
| *KANK2* | 0.84 |  | 0.19** | -0.10 | 0.04 | 0.18** |  | 0.17** | -0.05 |  | 0.84 |  | 0.19** | -0.11 | 0.03 | 0.17** |  | 0.16** | -0.04 |
| *COL6A1* | 0.83 |  | 0.18** | -0.07 | 0.06 | 0.18** |  | 0.20*** | -0.07 |  | 0.83 |  | 0.18** | -0.08 | 0.06 | 0.18** |  | 0.19** | -0.05 |
| *TRPC1* | 0.81 |  | 0.18** | -0.19** | 0.14* | 0.12 |  | 0.15** | -0.12 |  | 0.81 |  | 0.17** | -0.22*** | 0.15* | 0.11 |  | 0.13* | -0.10 |
| *COL16A1* | 0.83 |  | 0.17** | -0.18** | -0.02 | 0.13* |  | 0.17** | -0.05 |  | 0.83 |  | 0.17** | -0.20** | -0.02 | 0.13* |  | 0.16** | -0.05 |
| *COL3A1* | 0.83 |  | 0.17** | -0.12 | 0.06 | 0.13* |  | 0.16** | -0.13* |  | 0.83 |  | 0.17** | -0.14* | 0.06 | 0.13 |  | 0.14* | -0.12 |
| *RUNX1T1* | 0.86 |  | 0.16** | -0.02 | 0.07 | 0.20*** |  | 0.18** | -0.05 |  | 0.86 |  | 0.16** | -0.03 | 0.07 | 0.19** |  | 0.17** | -0.02 |
| *AEBP1* | 0.84 |  | 0.16** | -0.08 | 0.03 | 0.18** |  | 0.16** | -0.08 |  | 0.84 |  | 0.16** | -0.09 | 0.02 | 0.18** |  | 0.15* | -0.07 |
| *COL1A1* | 0.82 |  | 0.16** | -0.09 | 0.08 | 0.12 |  | 0.22*** | -0.07 |  | 0.82 |  | 0.16** | -0.10 | 0.08 | 0.11 |  | 0.21*** | -0.06 |
| *RGS11* | 0.81 |  | 0.13* | -0.08 | 0.05 | 0.15** |  | 0.24*** | -0.08 |  | 0.81 |  | 0.13* | -0.09 | 0.05 | 0.15* |  | 0.23*** | -0.06 |

* *P*-value < 0.1; ** *P*-value < 0.05; *** *P*-value < 0.01; MM: module membership; BW: birth weight; ME: module eigengene

**Table S8.** MM of hub genes and partial correlations of MEs and hub genes with maternal BMI and newborn birth weight variables before (n = 183) and after (n = 175) excluding mothers with gestational hypertension from the analysis.

| **Module eigengene/**  **Gene symbol** | **Main analysis** | | | | | | | | |  | **Excluding mothers with gestational hypertension** | | | | | | | | |
| --- | --- | --- | --- | --- | --- | --- | --- | --- | --- | --- | --- | --- | --- | --- | --- | --- | --- | --- | --- |
|  |  |  | **Maternal pre-pregnancy** | | | |  | **Newborn** | |  |  |  | **Maternal pre-pregnancy** | | | |  | **Newborn** | |
|  | **MM** |  | **BMI** | **Under-weight** | **Over-weight** | **Obese** |  | **BW** | **Low**  **BW** |  | **MM** |  | **BMI** | **Under-weight** | **Over-weight** | **Obese** |  | **BW** | **Low**  **BW** |
| ***Darkgray* module** | | | | | | | | | | | | | | | | | | | |
| **ME** |  |  | **0.15**** | **-0.05** | **-0.01** | **0.12** |  | **0.05** | **-0.19**** |  |  |  | **0.10** | **-0.04** | **-0.03** | **0.09** |  | **0.03** | **-0.16**** |
| *XLOC_000346* | 0.85 |  | 0.17** | -0.02 | 0.02 | 0.16** |  | 0.04 | -0.16** |  | 0.84 |  | 0.12 | -0.01 | 0.00 | 0.14* |  | 0.01 | -0.12 |
| *AHSP* | 0.88 |  | 0.17** | -0.05 | 0.00 | 0.12 |  | 0.04 | -0.18** |  | 0.88 |  | 0.11 | -0.03 | -0.03 | 0.09 |  | 0.03 | -0.13* |
| *XLOC_013489* | 0.92 |  | 0.17** | -0.01 | 0.01 | 0.14* |  | 0.02 | -0.18** |  | 0.92 |  | 0.12 | 0.00 | -0.01 | 0.12 |  | -0.01 | -0.15* |
| *SLC4A1* | 0.91 |  | 0.15** | -0.04 | -0.01 | 0.12 |  | 0.07 | -0.19** |  | 0.91 |  | 0.10 | -0.03 | -0.03 | 0.09 |  | 0.08 | -0.16** |
| *HBQ1* | 0.92 |  | 0.15** | -0.02 | -0.04 | 0.14* |  | 0.02 | -0.20*** |  | 0.91 |  | 0.11 | -0.01 | -0.06 | 0.11 |  | 0.00 | -0.17** |
| ***Darkred* module** | | | | | | | | | | | | | | | | | | | |
| **ME** |  |  | **0.14*** | **-0.03** | **0.05** | **0.15*** |  | **0.13*** | **-0.03** |  |  |  | **0.10** | **-0.03** | **0.03** | **0.13** |  | **0.12** | **-0.02** |
| *FZD4* | 0.83 |  | 0.24*** | -0.03 | 0.10 | 0.25*** |  | 0.20*** | -0.06 |  | 0.83 |  | 0.22*** | -0.02 | 0.08 | 0.24*** |  | 0.19** | -0.03 |
| *CDC42EP2* | 0.84 |  | 0.18** | -0.10 | 0.07 | 0.15* |  | 0.16** | -0.12 |  | 0.84 |  | 0.14* | -0.10 | 0.05 | 0.13 |  | 0.13* | -0.10 |
| *COL15A1* | 0.84 |  | 0.18** | -0.03 | 0.08 | 0.17** |  | 0.20*** | -0.08 |  | 0.85 |  | 0.16** | -0.03 | 0.05 | 0.16** |  | 0.20*** | -0.06 |
| *TBXA2R* | 0.81 |  | 0.18** | -0.05 | 0.12 | 0.13* |  | 0.16** | -0.02 |  | 0.82 |  | 0.14* | -0.04 | 0.10 | 0.11 |  | 0.17** | -0.01 |
| *EHD2* | 0.89 |  | 0.16** | -0.04 | 0.04 | 0.18** |  | 0.17** | -0.03 |  | 0.89 |  | 0.13* | -0.04 | 0.01 | 0.16** |  | 0.18** | -0.02 |
| *GPR124* | 0.89 |  | 0.16** | 0.00 | 0.05 | 0.18** |  | 0.22*** | -0.05 |  | 0.89 |  | 0.14* | 0.00 | 0.03 | 0.16** |  | 0.21*** | -0.04 |
| *VIM* | 0.90 |  | 0.15** | -0.03 | 0.12 | 0.16** |  | 0.17** | -0.05 |  | 0.90 |  | 0.12 | -0.03 | 0.09 | 0.14* |  | 0.17** | -0.04 |
| *EFEMP2* | 0.88 |  | 0.15** | -0.03 | 0.11 | 0.13* |  | 0.16** | -0.06 |  | 0.88 |  | 0.10 | -0.03 | 0.08 | 0.10 |  | 0.15* | -0.04 |
| *TBX2* | 0.83 |  | 0.12 | 0.02 | 0.07 | 0.19** |  | 0.18** | -0.07 |  | 0.84 |  | 0.10 | 0.02 | 0.03 | 0.17** |  | 0.19** | -0.07 |
| ***Gray60* module** |  |  |  |  |  |  |  |  |  |  |  |  |  |  |  |  |  |  |  |
| **ME** |  |  | **0.20***** | **-0.10** | **0.07** | **0.18**** |  | **0.14*** | **-0.04** |  |  |  | **0.18**** | **-0.10** | **0.04** | **0.17**** |  | **0.11** | **-0.02** |
| *COL8A2* | 0.88 |  | 0.24*** | -0.11 | 0.04 | 0.21*** |  | 0.16** | 0.01 |  | 0.88 |  | 0.25*** | -0.12 | 0.06 | 0.22*** |  | 0.14* | 0.02 |
| *MATN2* | 0.86 |  | 0.24*** | -0.09 | 0.10 | 0.19** |  | 0.18** | -0.09 |  | 0.86 |  | 0.25*** | -0.10 | 0.10 | 0.19** |  | 0.16** | -0.09 |
| *KANK2* | 0.84 |  | 0.19** | -0.10 | 0.04 | 0.18** |  | 0.17** | -0.05 |  | 0.81 |  | 0.16** | -0.19** | 0.12 | 0.11 |  | 0.13* | -0.11 |
| *COL6A1* | 0.83 |  | 0.18** | -0.07 | 0.06 | 0.18** |  | 0.20*** | -0.07 |  | 0.84 |  | 0.15** | -0.08 | 0.01 | 0.17** |  | 0.15* | -0.07 |
| *TRPC1* | 0.81 |  | 0.18** | -0.19** | 0.14* | 0.12 |  | 0.15** | -0.12 |  | 0.82 |  | 0.14* | -0.09 | 0.07 | 0.10 |  | 0.21*** | -0.06 |
| *COL16A1* | 0.83 |  | 0.17** | -0.18** | -0.02 | 0.13* |  | 0.17** | -0.05 |  | 0.84 |  | 0.17** | -0.07 | 0.04 | 0.17** |  | 0.19** | -0.07 |
| *COL3A1* | 0.83 |  | 0.17** | -0.12 | 0.06 | 0.13* |  | 0.16** | -0.13* |  | 0.84 |  | 0.15** | -0.10 | 0.02 | 0.15* |  | 0.16** | -0.03 |
| *RUNX1T1* | 0.86 |  | 0.16** | -0.02 | 0.07 | 0.20*** |  | 0.18** | -0.05 |  | 0.83 |  | 0.15** | -0.19** | -0.03 | 0.11 |  | 0.15* | -0.04 |
| *AEBP1* | 0.84 |  | 0.16** | -0.08 | 0.03 | 0.18** |  | 0.16** | -0.08 |  | 0.86 |  | 0.14* | -0.01 | 0.03 | 0.18** |  | 0.16** | -0.03 |
| *COL1A1* | 0.82 |  | 0.16** | -0.09 | 0.08 | 0.12 |  | 0.22*** | -0.07 |  | 0.83 |  | 0.15** | -0.12 | 0.04 | 0.12 |  | 0.15** | -0.12 |
| *RGS11* | 0.81 |  | 0.13* | -0.08 | 0.05 | 0.15** |  | 0.24*** | -0.08 |  | 0.81 |  | 0.12 | -0.08 | 0.01 | 0.14* |  | 0.22*** | -0.07 |

* *P*-value < 0.1; ** *P*-value < 0.05; *** *P*-value < 0.01; MM: module membership; BW: birth weight; ME: module eigengene

**Table S9.** Mediation analyses of the relationships among maternal pre-pregnancy BMI, expression of identified modules/hub genes, and newborn birth weight, using quasi-Bayesian Monte Carlo method with 10000 simulations.

| **Module eigengene/**  **Gene symbol** | **Direct effect** | | **Indirect effect** | | **%**  **mediated** |
| --- | --- | --- | --- | --- | --- |
|  | **Estimate (95% CI)** | ***P*** | **Estimate (95% CI)** | ***P*** |  |
| ***Darkgray* module** |  |  |  |  |  |
| **ME** | **13.09 (2.56, 23.55)** | **0.012** | **0.28 (-1.59, 2.38)** | **0.749** | **1.5** |
| *XLOC_000346* | 13.29 (2.74, 23.77) | 0.011 | 0.08 (-2.02, 2.24) | 0.932 | 0.5 |
| *AHSP* | 13.28 (2.73, 23.73) | 0.011 | 0.08 (-1.95, 2.21) | 0.930 | 0.5 |
| *XLOC_013489* | 13.51 (2.95, 23.91) | 0.009 | -0.14 (-2.24, 1.87) | 0.884 | -0.6 |
| *SLC4A1* | 12.91 (2.40, 23.33) | 0.013 | 0.47 (-1.32, 2.68) | 0.599 | 2.7 |
| *HBQ1* | 13.47 (2.92, 23.89) | 0.009 | -0.11 (-2.09, 1.80) | 0.905 | -0.4 |
| ***Darkred* module** |  |  |  |  |  |
| **ME** | **12.40 (1.94, 22.79)** | **0.016** | **0.98 (-0.50, 3.37)** | **0.239** | **6.2** |
| *FZD4* | 10.58 (0.01, 21.14) | 0.050 | 2.81 (0.21, 6.44) | 0.033 | 20.2 |
| *CDC42EP2* | 11.78 (1.28, 22.17) | 0.026 | 1.61 (-0.28, 4.49) | 0.110 | 11.0 |
| *COL15A1* | 11.22 (0.79, 21.64) | 0.034 | 2.17 (0.07, 5.32) | 0.038 | 15.3 |
| *TBXA2R* | 11.73 (1.28, 22.17) | 0.026 | 1.66 (-0.24, 4.56) | 0.098 | 11.4 |
| *EHD2* | 11.71 (1.27, 22.14) | 0.027 | 1.67 (-0.15, 4.54) | 0.084 | 11.5 |
| *GPR124* | 11.22 (0.89, 21.56) | 0.032 | 2.17 (0.04, 5.31) | 0.043 | 15.3 |
| *VIM* | 11.81 (1.42, 22.17) | 0.023 | 1.58 (-0.18, 4.38) | 0.091 | 10.8 |
| *EFEMP2* | 11.98 (1.58, 22.36) | 0.021 | 1.41 (-0.26, 4.10) | 0.125 | 9.5 |
| *TBX2* | 12.07 (1.77, 22.40) | 0.018 | 1.31 (-0.37, 3.94) | 0.149 | 8.7 |
| ***Gray60* module** |  |  |  |  |  |
| **ME** | **11.94 (1.42, 22.48)** | **0.025** | **1.44 (-0.68, 4.39)** | **0.191** | **9.9** |
| *COL8A2* | 11.38 (0.70, 21.98) | 0.036 | 2.01 (-0.56, 5.40) | 0.131 | 14.1 |
| *MATN2* | 11.00 (0.37, 21.51) | 0.042 | 2.38 (-0.11, 5.82) | 0.062 | 17.0 |
| *KANK2* | 11.44 (0.91, 21.84) | 0.031 | 1.94 (-0.11, 5.06) | 0.070 | 13.6 |
| *COL6A1* | 11.22 (0.82, 21.64) | 0.035 | 2.16 (0.08, 5.32) | 0.039 | 15.2 |
| *TRPC1* | 11.87 (1.36, 22.26) | 0.025 | 1.52 (-0.35, 4.34) | 0.127 | 10.3 |
| *COL16A1* | 11.69 (1.22, 22.11) | 0.027 | 1.69 (-0.16, 4.60) | 0.088 | 11.6 |
| *COL3A1* | 11.83 (1.40, 22.25) | 0.023 | 1.56 (-0.22, 4.36) | 0.106 | 10.6 |
| *RUNX1T1* | 11.66 (1.28, 22.08) | 0.027 | 1.73 (-0.11, 4.61) | 0.076 | 11.9 |
| *AEBP1* | 11.85 (1.36, 22.26) | 0.024 | 1.54 (-0.22, 4.34) | 0.106 | 10.5 |
| *COL1A1* | 11.21 (0.89, 21.54) | 0.033 | 2.18 (0.05, 5.35) | 0.042 | 15.4 |
| *RGS11* | 11.39 (1.19, 21.61) | 0.028 | 2.00 (-0.25, 5.16) | 0.083 | 13.9 |

Estimates (with 95% confidence intervals) represent the change in newborn birth weight (in gram) for a 1 kg/m^2^ increase in maternal pre-pregnancy BMI. The indirect effect represents the effect of maternal BMI on newborn birth weight that is mediated by the module eigengene (ME) or by the hub gene. The % mediated is the indirect effect divided by the total (direct + indirect) effect.
